# Observational evidence of overlooked downwelling induced by tropical cyclones in the open ocean

**DOI:** 10.1038/s41598-023-51016-0

**Published:** 2024-01-03

**Authors:** Chien-Yi Yang, Yiing Jang Yang, Yu-Heng Tseng, Sen Jan, Ming-Huei Chang, Ching-Ling Wei, Chuen-Teyr Terng

**Affiliations:** 1 Institute of Oceanography, National Taiwan University, Taipei, Taiwan; 2 Central Weather Administration, Taipei, Taiwan

**Keywords:** Physical oceanography, Atmospheric dynamics

## Abstract

Tropical cyclones (TCs) cause severe natural hazards and drive intense upper ocean cooling through a series of oceanic and atmospheric physical processes, including vertical mixing and upwelling. Among these processes, TC-induced warming of near-surface waters in the open ocean has rarely been noted. This study provides a detailed analysis of upper ocean responses to 30 TC events observed by two buoys in the western North Pacific between 2016 and 2021. Supplemented with numerical experiments, we suggest that downwelling frequently occurs at the periphery of upwelling regions (around the radius of the 34 knot wind speed) following the passage of a TC. Downwelling is identified via pronounced warm anomalies under a shallow mixed layer depth, and its dynamics are attributed to negative wind stress curl and current-induced convergence. These findings highlight the important role played by TC-induced downwelling and offer insights for reconsidering the influence of TCs on biogeochemical processes.

## Introduction

Tropical cyclones (TCs) are synoptic weather systems generated and intensified over the tropical and subtropical oceans. They may result in serious threats to human society on a global scale^1–3^. TC development is highly controlled by atmospheric and oceanic conditions; thus, a thorough understanding of TC-induced ocean responses and air–sea interactions is crucial for improving their forecasting^4–8^. Sea surface temperature plays a critical role in TC intensification through air–sea heat exchanges^4,9,10^. Furthermore, subsurface oceans temperatures can also significantly influence TC intensity^11–13^. A warmer upper ocean or warm eddies can provide significant air–sea fluxes, isolate cold water beneath the mixed layer to be upwelled to the near surface, strengthen TCs, and can even facilitate their rapid intensification^14–18^.

Vertical mixing and upwelling are the two primary oceanic processes responsible for changes in oceanic conditions following the passage of TCs in the open ocean. Strong TC winds force shear-induced vertical mixing in the upper ocean, resulting in the cooling of the near-surface layer of the ocean and warming of the subsurface layer^19,20^. Consequently, this deepens the mixed layer depth (MLD)^21,22^. Additionally, the positive wind stress curl within the radius of maximum wind speed (RMW) of TCs concurrently causes sea surface divergent flow, leading to upwelling of subsurface water and uplifting of the thermocline^20^. This combination of vertical mixing, upwelling, and air–sea heat exchanges contributes to a significant sea surface temperature decrease during and after the passage of TCs and generates the cold wake phenomenon^23–26^. TC-induced temperature decreases are pronounced in both the surface and subsurface layers, such that the magnitude of cooling is predominantly biased to the right–hand side of the TC track in the Northern Hemisphere^19^.

Satellite and in situ observations have revealed significant upper ocean heat loss induced by TCs via vertical mixing, upwelling, and air–sea heat fluxes^19,20,26–33^; however, few previous studies have noted warming in the subsurface or near-surface layers of the open ocean^33–37^. Shay et al*.* attributed this warming to the presence of convergent flows and downwelling processes^34,38^. Zhang et al*.* also found evidence for horizontal advection and downwelling as the dominant physical processes leading to warming anomalies associated with Typhoon Kalmaegi^39^. Inspired by these studies, we here aim to improve understanding of the physical processes involved in downwelling by analyzing an extensive dataset of TC observations measured by meteorological and oceanic data buoys.

**Figure 1:**
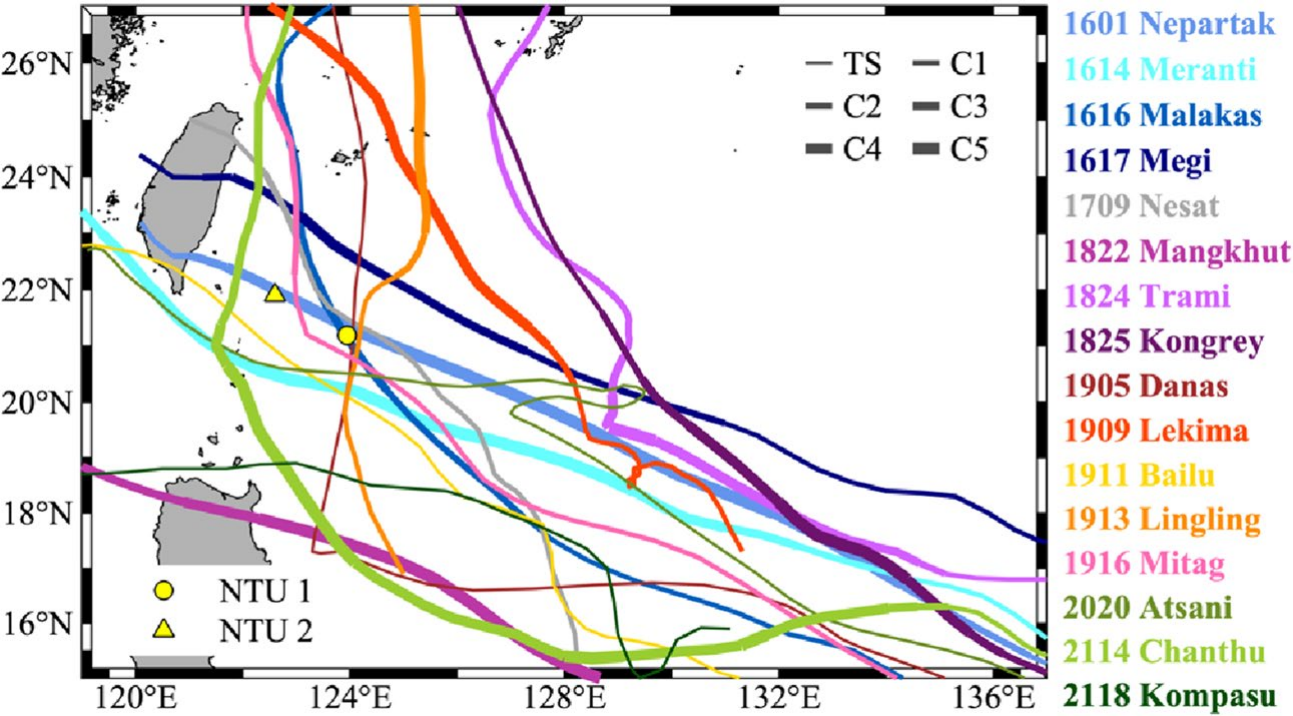
Track map of the sixteen TCs observed between 2016 and 2021 using IBTrACS data. IBTrACS data were obtained from http://www.ncdc.noaa.gov/ibtracs/^72,73^. The M_map package is used^82^. TC intensity is denoted by line thickness. The yellow circle and triangle denote the locations of the deployed buoys NTU1 and NTU2, respectively.

The western North Pacific is globally recognized as one of the most TC-active regions and is particularly noteworthy for generating some of the most intense and largest TCs recorded^40^. To better understand TC-induced upper ocean responses in the open ocean, two meteorological and oceanic data buoys were thus deployed to the southeast of Taiwan in the western North Pacific subtropical ocean; the two buoys were named NTU1 and NTU2 (see Materials and Methods)^41^. The distance between NTU1 (NTU2) and the southern tip of Taiwan was 375 (175) km. During the TC season (July–November) between 2016 and 2021, the two buoys captured data for sixteen TCs, including high-resolution data for the near-surface air and upper 500 m of the ocean (Fig. 1). A total of thirty TC events are included in this study, since some TCs were captured only by one of the two buoys. Notably, both buoys captured Super Typhoon Nepartak in July 2016, yielding unique findings complementary to previous studies^42^. The present study focuses on changes in the temperature profiles of the upper ocean during the aforementioned thirty events, which are indicative of the upwelling and downwelling processes resulting in heat gain (warming) and heat loss (cooling), respectively. A summary of the TCs studies and their corresponding oceanic parameters is presented in Table 1.

**Table 1:** Information for the sixteen TCs examined. DCPA is the closest distance between the TC track and the buoy, normalized by the ${{\text{R}}}_{34}$ value of the TC. Buoy 1 (2) indicates the NTU1 (NTU2) buoy. TFPA is the earliest time at which the buoy is within the ${1.5{\text{R}}}_{34}$ value of the TC.

No.	Name	Buoy	DCPA (${\text{R}}_{34}$)	TFPA (UTC)	Pre-TC MLD (m)	${\Delta {\text{UOHC}}}_{20\,^\circ{\rm C} }$ (kJ cm^−2^)
1601	Nepartak	1	0.08	2016-07-06 08:00	18.8	−54.7
		2	0.02	2016-07-06 14:00	19.5	−19.2
1614	Meranti	1	0.34	2016-09-12 18:00	59.2	+ 20.9
		2	0.45	2016-09-13 00:00	68.0	+ 14.2
1616	Malakas	1	0.01	2016-09-15 16:00	87.6	−38.8
		2	0.41	2016-09-16 01:00	100.1	−132.5
1617	Megi	1	0.39	2016-09-25 14:00	62.7	−30.8
		2	0.39	2016-09-25 21:00	59.8	−1.2
1709	Nesat	1	0.18	2017-07-27 22:00	34.0	−32.4
1822	Mangkhut	1	0.77	2018-09-13 21:00	37.5	−14.5
		2	0.85	2018-09-14 02:00	27.1	+ 75.4
1824	Trami	1	1.15	2018-09-25 12:00	42.7	+ 25.8
		2	1.28	2018-09-27 21:00	53.7	+ 20.7
1825	Kong-rey	1	1.17	2018-10-03 05:00	66.9	−28.1
		2	1.32	2018-10-03 16:00	72.6	−11.1
1905	Danas	1	0.08	2019-07-17 12:00	20.5	−25.1
		2	0.85	2019-07-17 13:00	41.8	+ 21.1
1909	Lekima	1	1.03	2019-08-07 06:00	24.4	+ 34.6
		2	1.08	2019-08-07 17:00	32.5	+ 18.2
1911	Bailu	1	0.45	2019-08-23 03:00	61.2	−3.6
		2	0.35	2019-08-23 12:00	70.7	−35.8
1913	Lingling	1	0.15	2019-09-02 20:00	53.3	−70.9
		2	1.16	2019-09-03 03:00	69.4	−20.1
1916	Mitag	2	0.16	2019-09-29 09:00	63.9	−77.3
2020	Atsani	1	0.32	2020-11-04 17:00	86.4	−25.5
		2	0.80	2020-11-05 09:00	87.8	−3.7
2114	Chanthu	1	0.94	2021-09-10 14:00	51.6	+ 19.6
		2	0.33	2021-09-10 15:00	47.8	−26.4
2118	Kompasu	1	1.05	2021-10-10 09:00	77.8	+ 3.5
		2	1.41	2021-10-10 12:00	54.0	+ 67.9

Upper ocean responses demonstrate a significant dependence on the intensity, size, relative position, and translation speed of TCs, as well as preexisting ocean stratification^8,19,43–45^. To gain an in-depth understanding of the processes by which the ocean responds to these conditions, the obtained dataset of the thirty TC events was carefully analyzed in the context of idealized, process-oriented numerical experiments to examine TC-forced ocean dynamics. On the basis of previous knowledge, we document the occurrence of open ocean downwelling in a significant number of TCs. Additionally, the radius of the 34 knot wind speed (${\text{R}}_{34}$) is used as a scaling factor to evaluate the distance of the buoy and TC track. The results of the observation datasets are preliminary categorized by ${\text{R}}_{34}$. Moreover, it is noted that upper ocean warming only indicates downwelling under certain hydrographic conditions, providing new insights into the relationship between upper ocean dynamics and TCs.

## Results

### TC-induced upper ocean warming

Two data buoys observed eleven out of thirty events from the sixteen TCs that showed TC-induced upper ocean warming (Table 1; Supplementary Figs. 3–32). These TCs passed sufficiently close to the buoys (${<1.5{\text{R}}}_{34}$) to affect local the ocean conditions. To investigate the impacts of these TC events on ocean conditions, we first examined pre- and post-TC conditions before and after the time to the first point of approach (TFPA). The TFPA is defined as the particular quadrant of ${1.5{\text{R}}}_{34}$ that first approaches the buoy, taking into account the orientation and symmetry of the TC wind field (see Materials and Methods). The durations of the pre- and post-TC conditions are two inertial periods (IPs) at the associated latitudes before and after TFPA, respectively. Upper ocean heat content (UOHC) was used as a thermodynamic variable to represent oceanic heat energy changes^46^. Negative (positive) UOHC anomalies were considered to represent heat loss or cooling (gain or warming) in the upper ocean after the passage of a TC. Among the eleven events exhibiting post-TC warming, the characteristics of each TC varied in terms of intensity (Tropical Storm to Category 5) and translation speed (see Supplementary Table 1). Considering this variability, further analyses are required to identify the factors controlling upper ocean warming.

**Figure 2:**
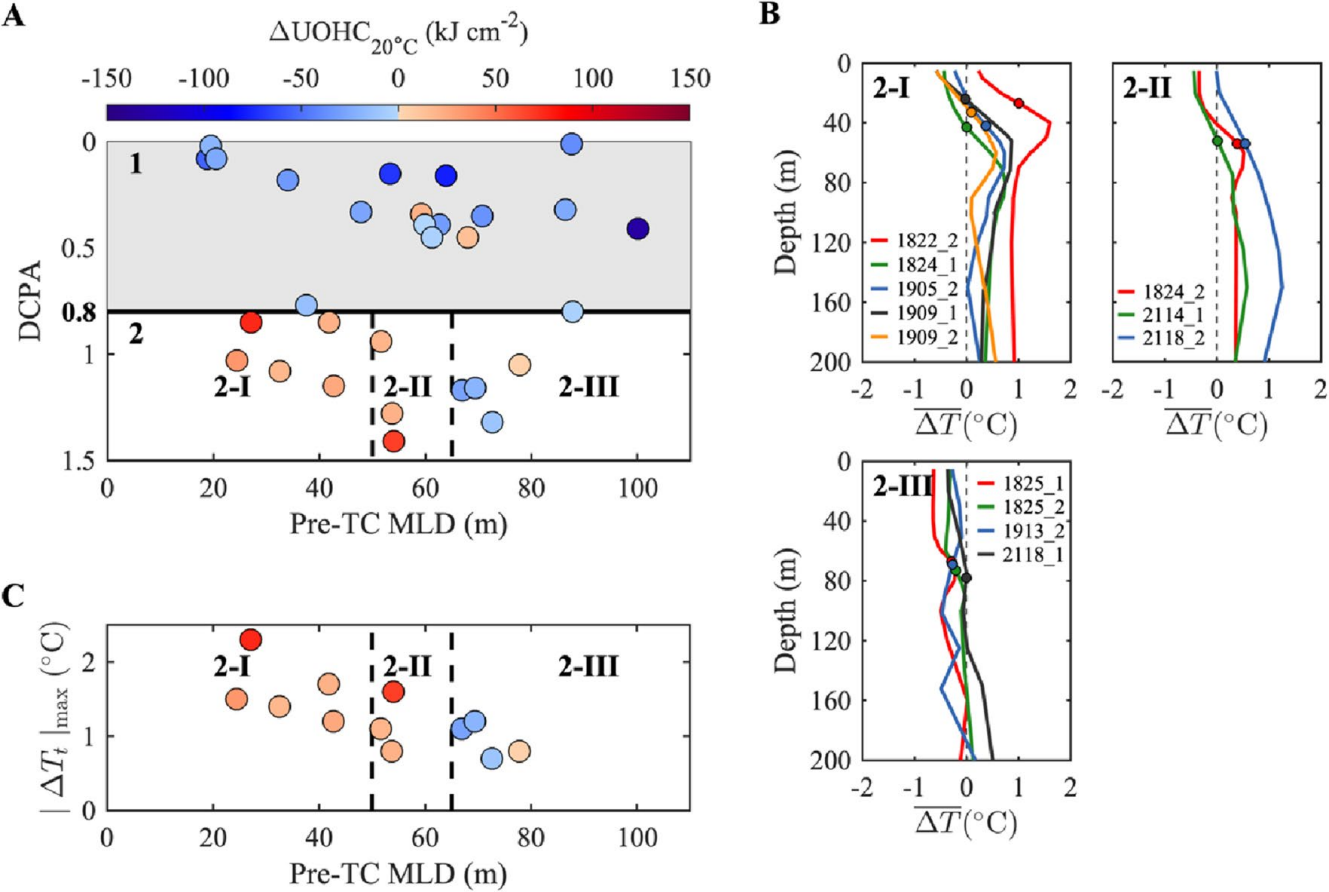
Pre- and post-TC changes of the thirty TC events and their relationships with distance and MLD. (**A**) Scatter plot showing the thirty TC events classified by pre-TC MLDs and DCPA. DCPA represents the closest distance between the TC track and the buoy, normalized by the specific ${\text{R}}_{34}$ value for each TC. The solid line distinguishes events into two groups: (1) DCPA ${\le 0.8{\text{R}}}_{34}$ and (2) DCPA ${>0.8{\text{R}}}_{34}$, with Group 1 shaded in grey. Subsequently, dashed lines further differentiate Group 2 into three distinct subgroups based on different pre-TC MLD ranges: (2-I) pre-TC MLD $\le 50 {\text{m}}$, (2-II) $50 {\text{m}}<$ pre-TC MLD $\le 65 {\text{m}}$, and (2-III) pre-TC MLD $>65 {\text{m}}$. Dot color indicates pre- and post-TC UOHC changes (${\Delta {\text{UOHC}}}_{20\,^\circ{\rm C} }$). (**B**) Vertical mean temperature anomaly profiles ($\overline{\Delta T}$) for events within subgroups 2-I, 2-II, and 2-III. The circle for each event represents the depth of its pre-TC MLD. (**C**) Scatter plot showing the relationship between pre-TC MLD and the maximum post-TC temperature anomaly (${\left|\Delta {T}_{t}\right|}_{{\max}}$) of events in Group 2. Dashed lines and colors are the same as those in (**A**).

TC-induced UOHC changes are dominated by the relative distance between the TC and the location of measurement, also known as the distance at the closest point of approach (DCPA); further influences from pre-TC ocean stratification have also been noted. For events occurring relatively close to the buoy (${\text{DCPA}}{\le 0.8{\text{R}}}_{34}$), observations showed that a majority were linked to heat loss (Fig. 2a). The only two close proximity events in which warming occurred were recorded in response to TC Meranti in 2016; these are unique cases that may have been influenced by the presence of a warm eddy in the local area at that time. Conversely, nine out of twelve events at greater distances from the TC tracks showed heat gain (${\text{DCPA}}>{0.8{\text{R}}}_{34}$). In addition, the disparity of preexisting ocean stratification, signified by the MLD, successfully distinguished the warming and cooling responses (Fig. 2a). Events with shallow pre-TC MLDs (≤65 m) exhibited warming; for example, a significant UOHC increase of 75.4 kJ cm^−2^ was observed at NTU2 in response to TC Mangkhut in 2018, comparable to other TC-induced heat losses (Table 1). Observations also suggest that analogous pre-TC MLDs could induce similar patterns of variation in vertical temperature structure (Fig. 2b). Five events with shallower pre-TC MLDs (≤50 m) resulted in a maximum temperature increase from 0.57 to 1.61 °C in the subsurface (Fig. 2b, I). In contrast, the vertical temperature anomaly was weaker (<0.5 °C) in events with deeper pre-TC MLDs (>65 m) (Fig. 2b, III). The maximum post-TC temperature anomaly in the upper ocean was found to be strongly inversely correlated with the pre-TC MLD (Fig. 2c). This relationship arises from the fact that a deeper pre-TC MLD hinders temperature change in the upper layers of the ocean. More specifically, vertical motions within this thick and temperature-uniform mixed layer are not capable of causing significant temperature change.

**Figure 3:**
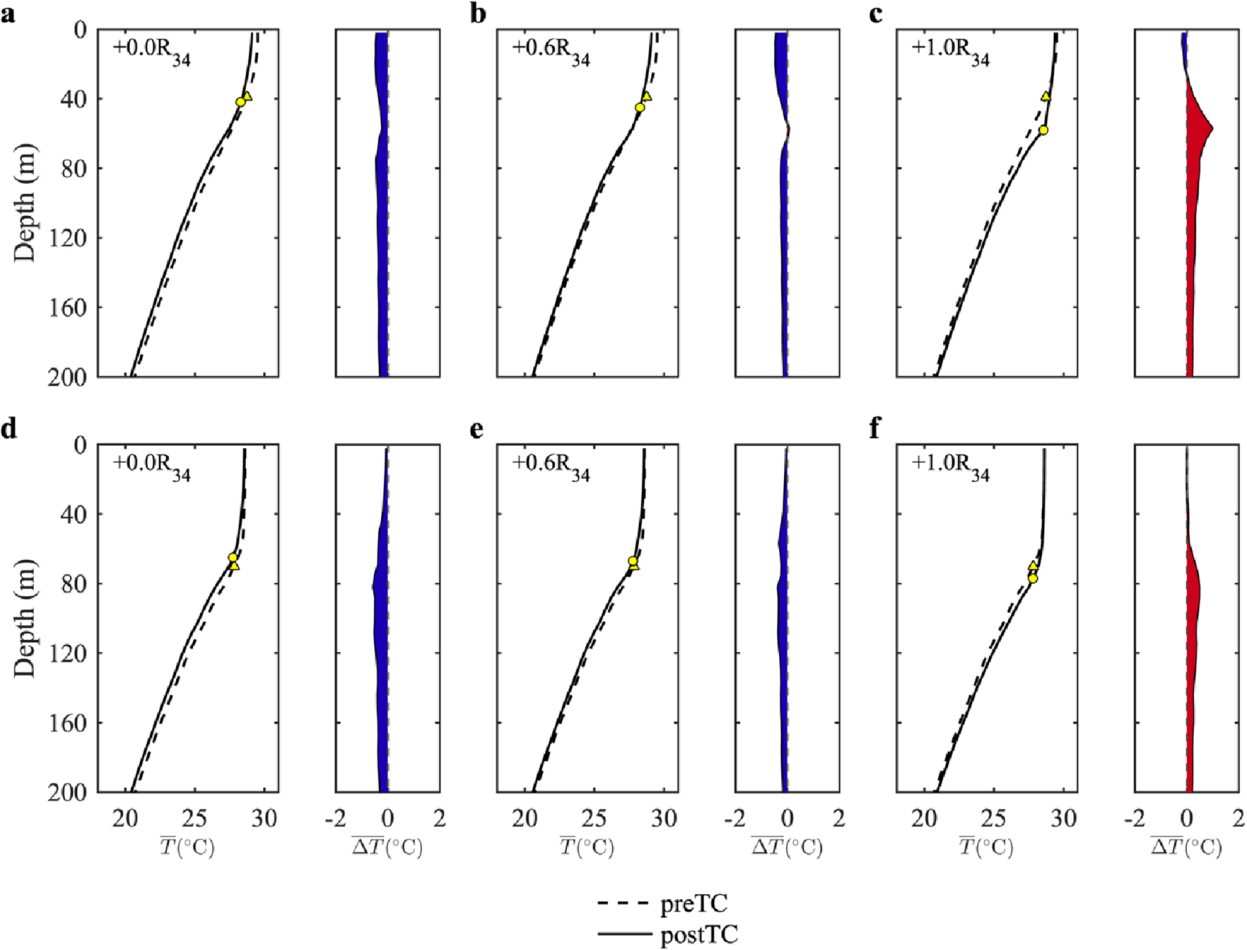
Simulated temperature profiles in the upper 200 m for two different MLD setups. Experiments were designated as Case A, a shallow MLD, and Case B, a thick MLD. Panels (**a**)–(**c**) show temperature profiles at different selected locations in Case A, whereas panels (**d**)–(**f**) depict the corresponding results for Case B. The dashed line represents pre-TC temperature, whereas the solid line represents post-TC temperature. Triangles and circles denote pre- and post-TC MLDs, respectively. In panels (**a**)–(**c**), Case A has a pre-TC MLD of 39 m, which subsequently changes to 42, 45, and 58 m. In panels (**d**)–(**f**), the pre-TC MLD of Case B is 70 m, with post-TC MLDs becoming 65, 67, and 77 m, respectively. The differences between the pre- and post-TC profiles are also shown, with blue (red) shading indicating a temperature decrease (increase).

Results from idealized, process-oriented numerical experiments using the TaIwan Multiscale Community Ocean Model (TIMCOM) were found to support the aforementioned dynamic analysis (see Materials and Methods)^47^. This study conducted two numerical experiments with different pre-TC MLDs, designated as Case A (relatively shallow) and Case B (relatively thick) to examine the dynamic role played by preexisting hydrographic conditions. Although the presence of an expected cold wake was distinguished at the surface, spatial variations in temperature anomalies within the subsurface layer were found to vary with crosstrack distance, displaying both cooling and warming anomalies (Supplementary Fig. 33). In these model results, we classify three distinct regions in the subsurface to distinguish the temperature responses. Firstly, in the vicinity of the TC track (${0{\text{R}}}_{34}$), the pattern of temperature decline within the subsurface was similar to that observed in the surface cold wake. Secondly, in the region around ${\pm 1{\text{R}}}_{34}$, two band-like warming regions were observed parallel to the track, with the magnitude of the temperature anomaly being comparable to that of the surface cold wake. Thirdly, at approximately ${\pm 0.6{\text{R}}}_{34}$, alternating warming and cooling anomalies occurred in near-inertial oscillations along the TC orientation. Correspondingly, the vertical structures of the temperature variation at ${0{\text{R}}}_{34}$, ${\pm 0.6{\text{R}}}_{34}$, and ${\pm 1{\text{R}}}_{34}$ suggested different responses in the temperature profile at different distances (Fig. 3). Additionally, Fig. 3 demonstrates that the pre-TC MLD significantly influences the strength of temperature anomalies in the warming band, as corroborated by our observational analyses. The temperature anomalies observed in cases A and B demonstrated obvious differences, particularly in regions distant from the TC track. At ${\pm 1{\text{R}}}_{34}$, subsurface warming was more pronounced in Case A than that in Case B (Fig. 3f). In accordance with the observational results (Fig. 2c), the comparison of numerical results for cases A and B supported the hypothesis that thicker water in the mixed layer inhibits the temperature changes induced by vertical motions.

### Distinct physical processes related to the warming

**Figure 4:**
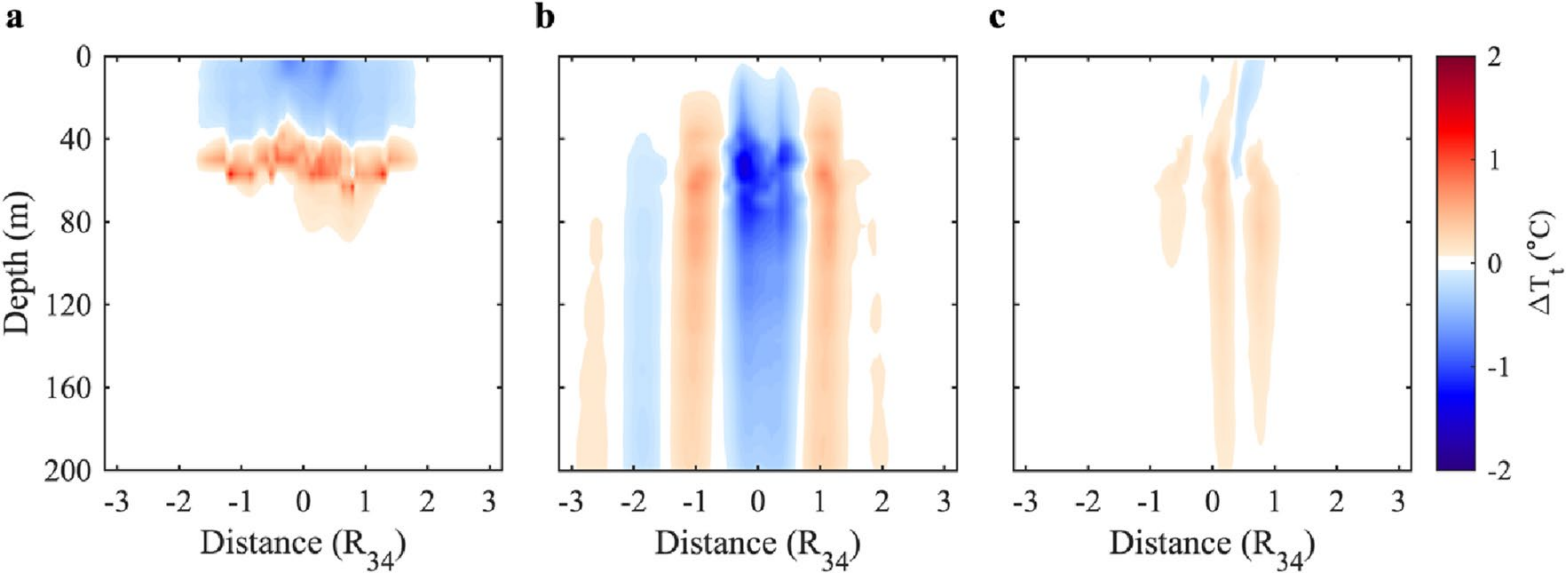
Depth-crosstrack section of $\Delta {T}_{t}$ for the numerical experiment Case A. The temperature anomaly may be attributed to (**a**) vertical mixing, (**b**) vertical advection, and (**c**) horizontal advection.

Distinct physical processes, such as vertical mixing, vertical advection, and horizontal advection, play a significant role in TC-induced temperature changes. To gain an understanding of the contributions of these physical processes, analyzing temperature profile changes can provide preliminary insights. In a mixing-dominated scenario, near-surface cooling and subsurface warming values offset each other. However, when considering both mixing and upwelling (downwelling), the temperature profile shifts upward (downward)^19,20,48^. Another method applicable to our model experiment results is heat budget analysis (see Materials and Methods). To gain an understanding of the individual contributions of these physical processes, heat budget analysis may be applied (see Materials and Methods). Both cases A and B exhibited downwelling at depths slightly below those of the preexisting MLDs (Fig. 4 & Supplementary Fig. 35). Here, we will focus on presenting the results of Case A, which had a shallower pre-TC MLD.

Figure 4a suggests that, following the passage of TC, vertical mixing mainly induced temperature variation around the track, with a slight bias of temperature anomalies to the right; this could be a feature of the rightward bias whereby stronger inertial currents occur on the right-hand side of the track in the Northern Hemisphere (Fig. 4a). Under the effect of vertical mixing, the total extent of subsurface layers with warming anomalies was approximately equivalent to the extent of near-surface layers exhibiting cooling. This implies a net vertical balance between warming and cooling effects as a result of vertical mixing. Upwelling also caused a temperature decrease around the track with a smaller crosstrack distance (Fig. 4b). Importantly, at the periphery of the net cooling regions caused by upwelling, downwelling contributed to a noticeable temperature increase and created warming bands on both sides of and parallel to the track. The position of the downwelling-induced warming band range from approximately ${0.8{\text{R}}}_{34}$ to ${1.5{\text{R}}}_{34}$ (Fig. 4b), which is in agreement with the observed DCPA values of the eight warming events (Fig. 2a, I and II), which lie within ${0.85{-}1.41{\text{R}}}_{34}$ (Table 1). In comparison with the upwelling-induced cold anomalies, downwelling-induced warm anomalies were weaker but more widespread^36,39^. Horizontal advection could also cause weak positive temperature anomalies, reducing the overall cold anomalies in upwelling-dominated regions. According to heat budget analysis, vertical mixing and upwelling both strengthen the well-documented upper ocean cooling. Notably, we confirm that downwelling, although less widely discussed than upwelling, is also an influential post-TC physical process for TC-induced temperature variation.

### Key role of downwelling

**Figure 5:**
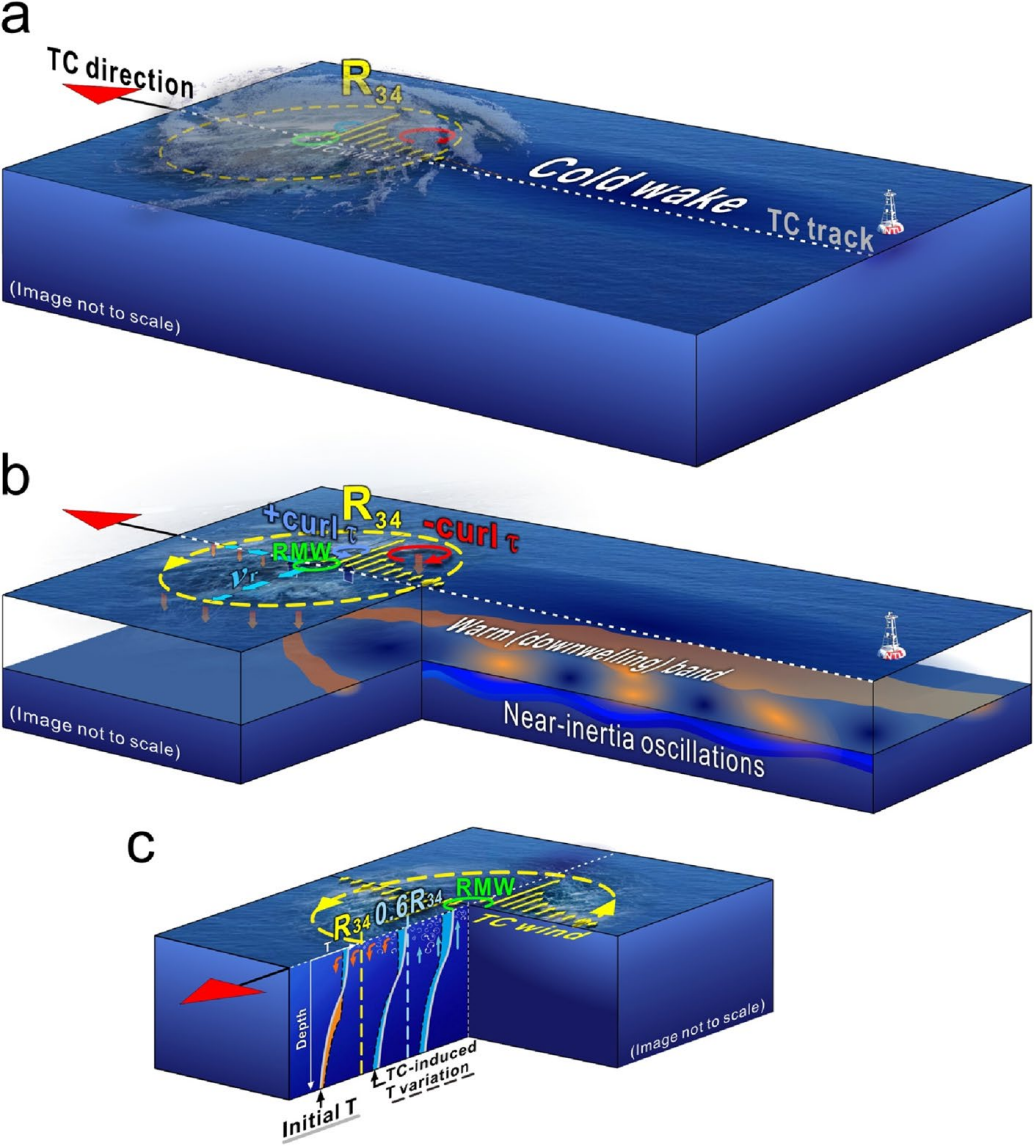
Schematic diagram of TC-induced upper ocean responses and processes in different layers. (**a**) Temperature responses at the sea surface. (**b**) Temperature and current responses in the surface and subsurface layers. Yellow dashed circle indicates a distance of $1{\text{R}}_{34}$ from the TC center, while green solid circle marks the RMW. Yellow vectors represent the TC wind field. Inside (outside) the RMW, positive (negative) wind stress curl is indicated by blue (red) circles, resulting in upwelling (downwelling) denoted by upward blue (downward red) arrows. Downwelling caused by horizontal convergence is marked by downward red arrows adjacent to $1{\text{R}}_{34}$. (**c**) Depth versus crosstrack section with three profiles at different crosstrack locations, i.e., $0{\text{R}}_{34}$, ${0.6{\text{R}}}_{34}$, and ${1{\text{R}}}_{34}$. Orange and blue shading represents warming and cooling, whereas orange and blue arrows indicate downwelling and upwelling, respectively.

Our analyses reveal that temperature responses after the passage of a TC are driven by a complex interplay of physical processes. As schematically illustrated in Fig. 5, a pronounced cold wake generally occurs at the surface following the passage of a TC (Fig. 5a). Figure 5b shows that the motion of a translating TC generates inertial pumping in its lee through alternating patterns of divergence and convergence at near-IPs, thereby leading to oscillating downwelling and upwelling^34,38,43,49–51^. The locations of these oscillations are related to the horizontal current field driven by a rotating wind. It is important to note that integrating through an IP may result in a compensatory effect on the net temperature response caused by inertial pumping, whereby cooling occurs during upwelling and warming occurs during downwelling.

Additionally, we specifically emphasis on the spatial distribution of vertical motions and discuss the contributing factors of the downwelling processes (Fig. 5b, Supplementary Fig. 33). Ekman pumping is an essential mechanism causing upward (downward) motion via positive (negative) wind stress curl^49,52^. Due to the spatial distribution of wind stress, downwelling outside the RMW is typically weaker than upwelling inside the RMW, explaining the preferential documentation of upwelling in previous studies. It is worth noting that when TCs translate more rapidly, the upper ocean response based on Ekman’s theory is weaker due to an insufficient response time to generate upwelling and downwelling processes^53,54^.

The occurrence of downwelling is also related to the convergence of strong TC wind-induced currents encountering relatively slow-moving ocean currents under the constraints of continuous flow. Comparing the model-produced vertical velocity with analytical solutions of Ekman pumping, it suggests that the contribution of Ekman pumping is not adequate to account for the entire downward velocity output, potentially indicating the presence of flow convergence-induced downwelling. This downwelling can result in warming anomalies at both edges of the TC’s left and right quadrants^34,38^. Temperature increases persist at the front of the TC after translation; however, this convergence-induced warming is not evident adjacent to the TC track due to its weakness in comparison to the subsequent cooling of the cold wake. In contrast, this weak temperature increase might be more persistent at greater distances from the TC track (Supplementary Fig. 34).

Each of the driving processes previously discussed appears in different regions. Oscillatory downwelling and upwelling modulates temperature changes in the TC-forced region. This vertical oscillation is known to have a negligible impact on local temperature changes during IPs because both the cooling effect of upwelling and the warming effect of downwelling tend to compensate for the other. Thus, the spatial distributions of TC-induced temperature responses during IPs are determined by a combination of Ekman pumping and convergence, as well as vertical mixing, with the exception of vertical oscillation (Fig. 5c).

In the context of Ekman pumping, the presence of positive (negative) wind stress curl corresponds to upwelling (downwelling) inside (outside) the RMW. Horizontal convergence-induced downwelling typically occurs adjacent to $\pm 1{\text{R}}_{34}$, where the maximum gradient of horizontal surface currents is found. Wind-driven vertical mixing, is primarily associated with regions of strong wind force, which is significant inside the ${\text{R}}_{34}$. In summary, cooling near the TC center is mainly dominated by upwelling and vertical mixing, whereas the majority of warming events are observed within the range of ${0.8{\text{R}}}_{34}$ to ${1.5{\text{R}}}_{34}$, consistent with observational results (Fig. 2a). Accordingly, ${\text{R}}_{34}$ is useful for scaling the crosstrack distance and quantifying the spatial distribution of downwelling-induced warming, whereas RMW provides a better measure of the cold wake phenomenon^19^.

## Discussion

The observational analysis presented in this study has revealed the presence of open-ocean downwelling induced by a significant number of TCs. It should be emphasized that prominent TC-induced downwelling generally occurs during and following the passage of TCs and that the identification of upper ocean warming is primarily determined by two factors: (i) proximity of the observation location to the TC track (scaled by ${\text{R}}_{34}$), such that warming could only be observed at the periphery of the cold wake, and (ii) preexisting MLDs, wherein the thick-mixed layer could inhibit temperature changes resulting from downwelling. In addition to the observational evidence, idealized numerical simulations support our inference, suggesting that although the cold wake justly receives much attention, the accompanying downwelling process is important and warrants further investigation.

Numerical model simulated TC-induced warming and downwelling are consistent with the features identified from observations. Although the process-oriented numerical experiments in this study primarily provided qualitative analysis, the numerical schemes and model settings, which included detailed ocean–atmosphere interactions and surface heat fluxes, and the resolving of mesoscale eddies, can also realistically affect the results of the simulations^8,19,43–45^. Although the translation speeds of all observed TC events in this study exceeded the speed of the first baroclinic mode in the ocean^41^, translation speed remains a crucial factor for determining the ocean's response to TCs. In contrast to fast-moving TCs, slow-moving TCs are more responsive to upwelling, which occurs instantly behind the TC center and interacts with vertical mixing^19,49,53^. That is to say, our numerical experiments were insufficient to cover all of the situations represented by the thirty TC events, but nonetheless provide an essential explanation for the dynamics behind the observational results. In addition to our model experiments, we have supplemented with realistic simulations from the Global Ocean Forecasting System^55,56^. These simulations consider complex physical conditions and display temperature increases in the subsurface in some selected events (Supplementary Figs. 36, 37). Undoubtedly, more detailed information and in situ observations will be necessary in the future to further improve our understanding of TC-induced downwelling.

In summary, we investigated the existence and dynamics of TC-induced downwelling in the open ocean. Using temperature variation as an indicator of downwelling, we emphasized that the location chosen for observation after the passage of TC has a crucial impact on determining the dominant physical process and the resulting temperature response (Figs. 2 and 5). Preexisting stratification is therefore verified as a key parameter in determining responses, such as that a shallower preexisting MLD favors the occurrence of upper ocean warming over a thicker MLD. Negative wind stress curl-induced Ekman pumping and surface flow convergence are the two major processes causing downwelling during and after a TC. This presents a broad view of the possible changes in the thermal structure of the upper ocean in the aftermath of a TC, which could in turn significantly impact oceanic preconditioning and subsequently affect the intensity of upcoming TCs^57,58^. Coupled ocean–atmosphere models improve TC forecasts significantly by incorporating the ocean impact into the model, which is particularly notable in slow-moving TC cases^59,60^. Reevaluating the downwelling process in the ocean could also benefit the parameterization of numerical models for TC forecasting. Furthermore, our study also offers an opportunity to reassess the impact of TCs on biological and biogeochemical processes in the upper ocean, including carbon and nutrient fluxes and the distribution and structure of biological communities^61–71^, which aligns with the objectives of the United Nations Decade of Ocean Science for Sustainable Development 2030.

## Methods

### Buoy configuration

In situ oceanic and atmospheric data were collected using moored surface buoys^41^. Two buoys were deployed each summertime from 2016 to 2021 at 123.9 °E and 21.1 °N (NTU1) and 122.6 °E and 21.9 °N (NTU2). The buoys measured wind, air temperature, air pressure, relative humidity, precipitation, solar radiation, and the height of waves above the sea surface (Supplementary Fig. 1). Underwater devices including conductivity-temperature-depth sensors, temperature–pressure sensors, and current meters were also used (Supplementary Fig. 2). Although the vertical resolutions of the underwater devices varied each year, high-resolution temperature data with intervals no greater than 25 m were obtained throughout the upper 150 m. The time intervals of each sensor ranged from 1 to 6 min.

### Tropical cyclone data

Data for tropical cyclones (TCs), including position, intensity, translation speed, the radius of the 34 knot wind speed (${\text{R}}_{34}$), and the radius of maximum wind speed (RMW), were obtained from the International Best Track Archive for Climate Stewardship (IBTrACS; 72, 73). The main data source chosen in this archive included datasets retrieved from the Joint Typhoon Warning Center (JTWC). It is important to note that IBTrACS provides ${\text{R}}_{34}$ data in each quadrant (NE, SE, SW, and NW), and that the calculation of ${\text{R}}_{34}$ values is given by weighting the relative angle of TCs and buoys. This approach was essential for defining the asymmetric ${\text{R}}_{34}$ wind field.

### Buoy data analysis

TC arrival time, also known as the time to the first point of approach (TFPA), is defined as the time at which ${1.5{\text{R}}}_{34}$ first approached the buoy. To eliminate differences in TC size, the distance at the closest point of approach (DCPA) was normalized using the mean value of ${\text{R}}_{34}$ from TFPA to the closest point of approach.

For the analysis presented herein, we used vertically interpolated temperature data in from ${\text{TFPA}}-2{\text{IP}}$ to ${\text{TFPA}}+2{\text{IP}}$, which data before TFPA indicate pre-TC conditions and data after TFPA indicate post-TC conditions. This temperature timeseries is denoted by ${T}_{t}$, with the subscript $t$ indicating time from $-2{\text{IP}}$ to $+2{\text{IP}}$. Herein, pre-TC profile refers to the temperature profile ($\overline{{T}_{i}}$) calculated as the average temperature from -$2{\text{IP}}$ to TFPA using the equation:

$$\overline{{T}_{i}}= {\sum }_{t=-2{\text{IP}}}^{t={\text{TFPA}}}{T}_{t} / 2{\text{IP}}.$$

Similarly, the mean profile of post-TC temperature data is given by:

$$\overline{T}= \sum_{t={\text{TFPA}}}^{t=+2{\text{IP}}}{T}_{t} / 2{\text{IP}}.$$

The overlines in $\overline{{T}_{i}}$ and $\overline{T}$ indicate that both variables are one-dimensional vertical profiles. We then calculated the timeseries of temperature difference by subtracting the initial state ($\overline{{T}_{i}}$) from the temperature timeseries (${T}_{t}$) as follows:

$$\Delta {T}_{t}={T}_{t}-\overline{{T}_{i}}, t= -2{\text{IP}}\sim +2{\text{IP}}.$$

This difference between the post-TC and pre-TC mean profile is expressed as follows:

$$\overline{\Delta T}= \overline{T}- \overline{{T}_{i}}.$$

To exclude the influence of semidiurnal and diurnal tides and other high-frequency noises, a 28-h low-pass filter was applied to the raw data considering IPs of 33.24 and 32.08 h at the latitudes of NTU1 and NTU2, respectively.

### UOHC calculation

Following Chandra and Kumar^74^, the UOHC can be expressed as follows:

$${\text{UOHC}}_{20\,^\circ {\text{C}}}={\int }_{z\left(T=20\,^\circ {\text{C}}\right)}^{\text{surface}}\rho {C}_{p}\left(T-20\right) dz,$$

where $\rho (= 1026\,{\text{kg\,m}}^{-3})$ is seawater density, ${C}_{p}$ (= 4178 J kg °C^−1^) is specific heat capacity, and $T$ is temperature. ${\Delta {\text{UOHC}}}_{20\,^\circ {\text{C}}}$ is defined similarly to $\overline{\Delta T}$, with the exception of that ${\text{UOHC}}_{20\,^\circ {\text{C}}}$ represents a mean value since the timeseries of ${\text{UOHC}}_{20\,^\circ {\text{C}}}$ is not provided in this study. Typically, UOHC is calculated by integrating the temperature from the surface to the depth of the $26\,^\circ{\rm C}$ isotherm, which makes a significant contribution to the TC intensity^75^. Conversely, the possible impact of TC-induced ocean responses might be deeper than the 26 °C isotherm^30,31,33^, thus the ${\text{UOHC}}_{20\,^\circ {\text{C}}}$ may better represent the ocean thermal field in this study. Moreover, the vertical extent of the figures in this study is limited to 200 m since the $20\,^\circ{\rm C}$ isotherm is at approximately at 200 m in the observational data.

### Numerical model

The TaIwan Multi-scale Community Ocean Model (TIMCOM) is a three-dimensional, free-surface model used to study oceanic responses to an idealized, constantly translating TC^47^. Therefore, an idealized TC was simulated in a homogeneous and motionless ocean, moving from east to west at a constant speed ($6\,{\text{ms}}^{-1}$) that exceeded the baroclinic wave speed, resulting in the formation of a wake generated by the TC^43^. The constant speed of the TC was determined based on the average translation speed of the 16 TCs, most of which translated at a moderate speed ($5{-}7\,{\text{ms}}^{-1}$), i.e., faster than the slow translation speed ($2{-}3\,{\text{ms}}^{-1}$) (Table S1)^76^. The simulation lasted for 8 days, using a time step of 75 s. The model domain was 6000 km in the zonal direction and 1200 km in the meridional direction, and the horizontal grid space was 10 km in both directions. The thickness of the water column was 5000 m; specifically, the vertical resolution in the upper 100 m of the water column was 2, 8, 14, 20, 26, 32, 38, 44, 50, 57, 63, 69, 75, 82, 88, and 95 m.

Wind stress ($\tau$) was determined using a modified Rankine vortex with linear functions and a maximum value of ${\tau }_{{\max}}=2\,{\text{Nm}}^{-2}$; the process is given by the following expression:

$$\tau \left(r\right)= {\tau }_{{\max}} \frac{r}{\text{RMW}} for r\le {\text{RMW}},$$

$$\tau \left(r\right)={\tau }_{{\max}} \frac{r-{r}_{{\max}}}{{\text{RMW}}-{r}_{{\max}}} for {r}_{{\max}}>r>{\text{RMW}},$$

$$\tau \left(r\right)=0 for r\ge {r}_{{\max}},$$

where r is distance from the TC center, RMW is the radius of maximum wind speed, and ${r}_{{\max}}$ is the maximum radius of the TC where $\tau$ is 0^77^. In this study, RMW and ${r}_{{\max}}$ were set at 60 and 240 km, respectively, to yield an approximate value of 188 km, denoted as ${\text{R}}_{34}$.

Surface heat fluxes and radiation were set to zero for the sake of simplification and salinity was maintained constant throughout the experiment. To parameterize vertical mixing, both the Price-Weller-Pinkel and Pacanowski and Philander (PP82) schemes were adopted^78,79^.

To investigate the effects of preexisting MLDs on the simulation results, we designed two experiments with relatively shallow (Case A) and relatively thick (Case B) preexisting MLDs. The preexisting MLD of Case A is 39 m and the preexisting MLD of Case B is 70 m.

### Heat budget calculation

The heat budget can be obtained as follows:

$$\frac{\partial T}{\partial t}=-\left(u\frac{\partial T}{\partial x}+v\frac{\partial T}{\partial y}\right)+\left(-w\frac{\partial T}{\partial z}\right)+\left({\kappa }_{z}\frac{{\partial }^{2}T}{\partial {z}^{2}}\right),$$

where $u$ and $v$ are the zonal and meridional current components, respectively, $w$ is vertical velocity, and ${\kappa }_{z}$ is vertical eddy diffusivity^80^.

### MLD

The MLD was determined by a temperature-based criterion of $0.8\,^\circ{\rm C}$ absolute difference from a reference depth at 10 m^81^.

### Supplementary Information

Supplementary Information.

## Data Availability

Data needed to evaluate our findings in the paper are available at: 10.5281/zenodo.7789044.
