# Supplementary material for: Observational evidence of overlooked downwelling induced by tropical cyclones in the open ocean
Source: Sci Rep. 2024 Jan 3;14:335. doi: 10.1038/s41598-023-51016-0 (PMC10764306; doi:10.1038/s41598-023-51016-0)
Supplement: Supplementary file 1 — Supplementary Information. [file 41598_2023_51016_MOESM1_ESM.pdf]

Supplementary Information

Observational Evidence of Overlooked Downwelling Induced by Tropical Cyclones in the Open Ocean

Chien-Yi Yang,[1] Yiing Jang Yang,[1]* Yu-Heng Tseng,[1] Sen Jan,[1] Ming-Huei Chang,[1] Ching-Ling Wei,[1] Chuen-Teyr Terng[2]

[1] Institute of Oceanography, National Taiwan University, Taipei, Taiwan.
[2] Central Weather Administration, Taipei, Taiwan.
* Corresponding author. Email: yjyang67@ntu.edu.tw

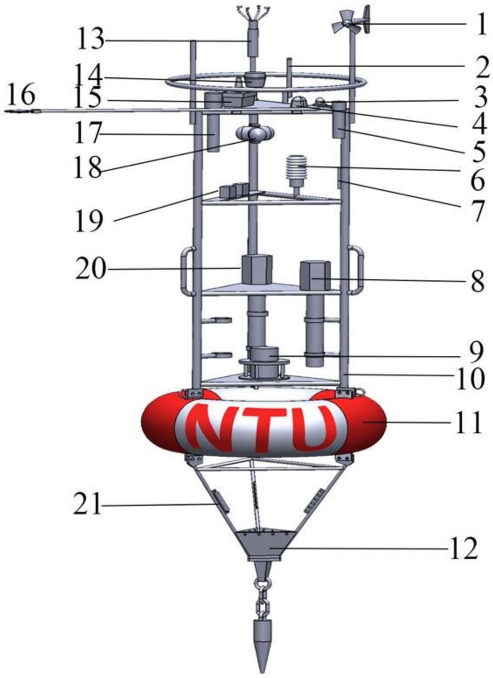

2021

ITEM	DESCRIPTION
1	Propeller anemometer
2	UHF antenna
3	Pyrometer
4	Inmarsat satellite antenna
5	Iridium GPS beacon
6	Air temperature and humidity probes
7	Barometer
8	Control and communication unit
9	Wave gauge
10	Tower
11	Fiberglass buoy
12	Bridle
13	Ultrasonic anemometer
14	Solar marine navigation light
15	Iridium satellite antenna
16	Net radiometer
17	Rain gauge
18	Radar reflector
19	Time-lapse camera x 2
20	Meteorological data acquisition unit
21	Sea surface temperature

Supplementary Figure 1. Schematic of the meteorological instruments installed on the buoys. The 2021 design is used as an example case.

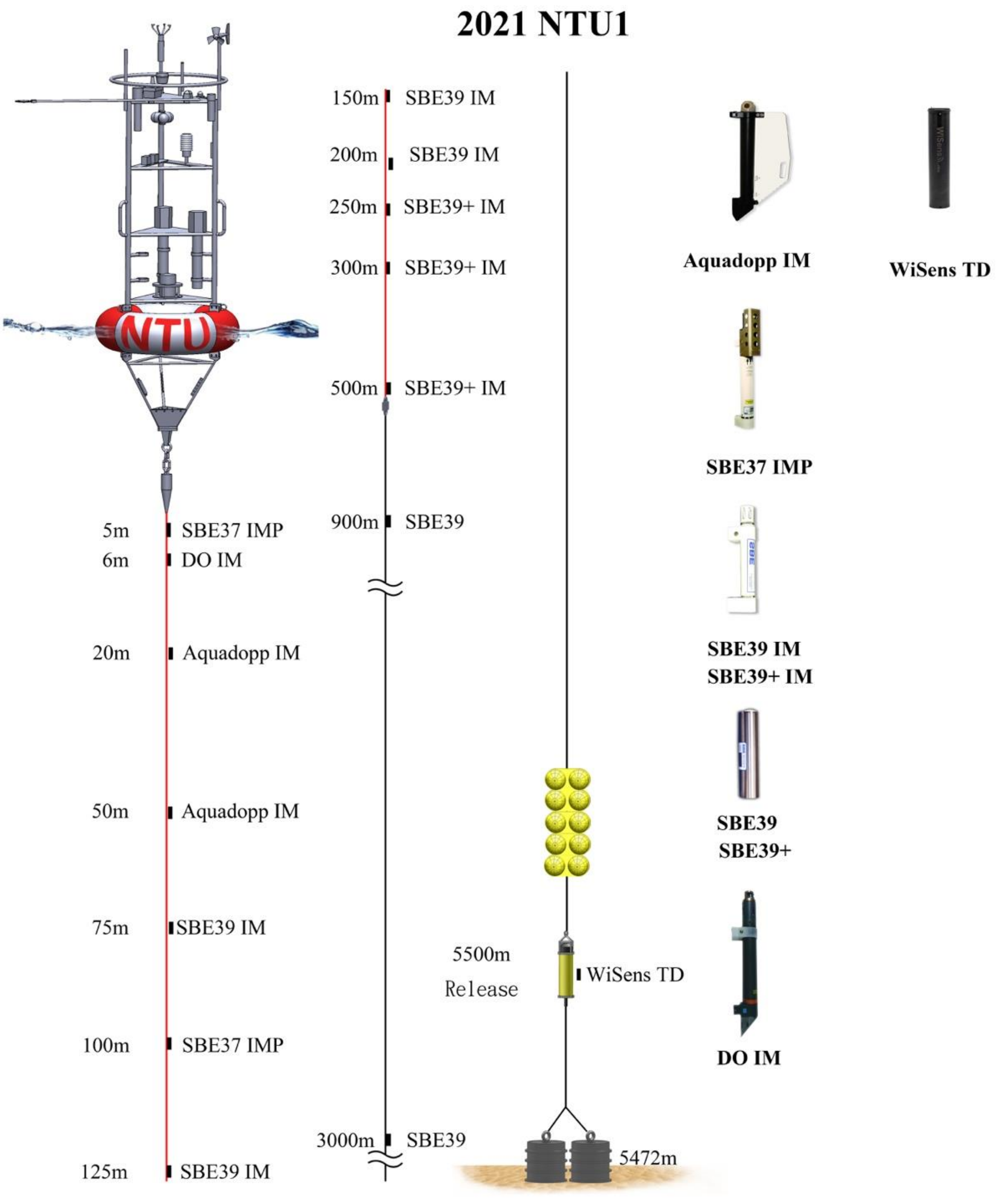

Supplementary Figure 2. Schematic of the mooring diagram and underwater instruments associated with the buoy. The 2021 design is used as an example case.

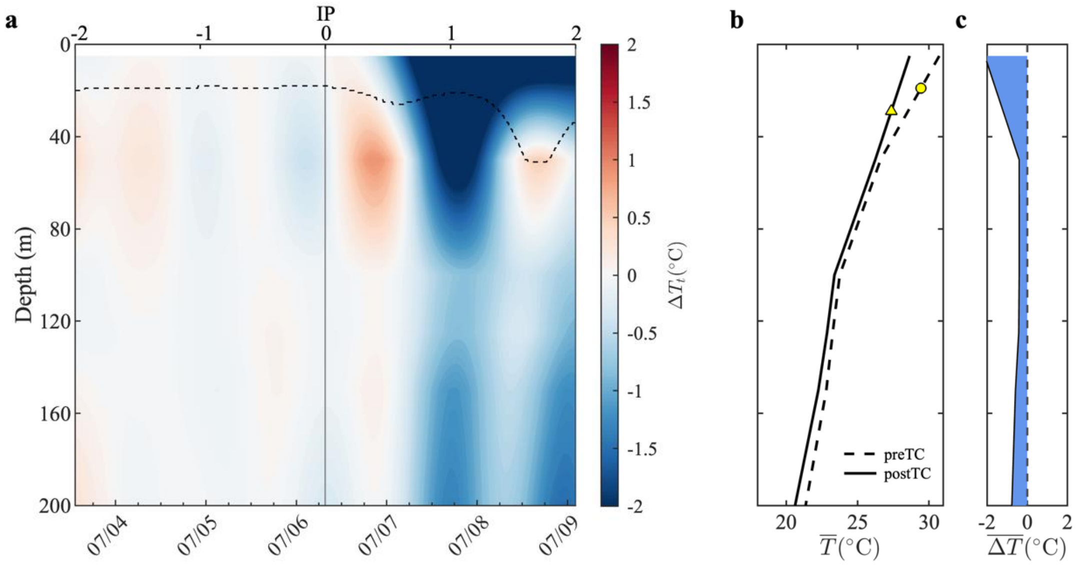

Supplementary Figure 3. Temperature data of Nepartak 1. (**a**) Depth–time contours $\boldsymbol{\Delta T}$ in the upper 200 m during approximately two IPs before and after TFPA. Vertical black solid line indicates TFPA and black dashed line denotes the MLD. (**b**) Pre-TC (dashed line) and post-TC (solid line) temperature profiles are shown for the upper 200 m, with the circle (pre) and the triangle (post) denoting the MLD. Pre- and post-TC profiles are the mean temperatures over two IPs before and after TFPA, respectively. (**c**) The difference between the pre- and post-TC profiles is presented. Blue shading denotes a temperature decrease; red shading denotes a temperature increase.

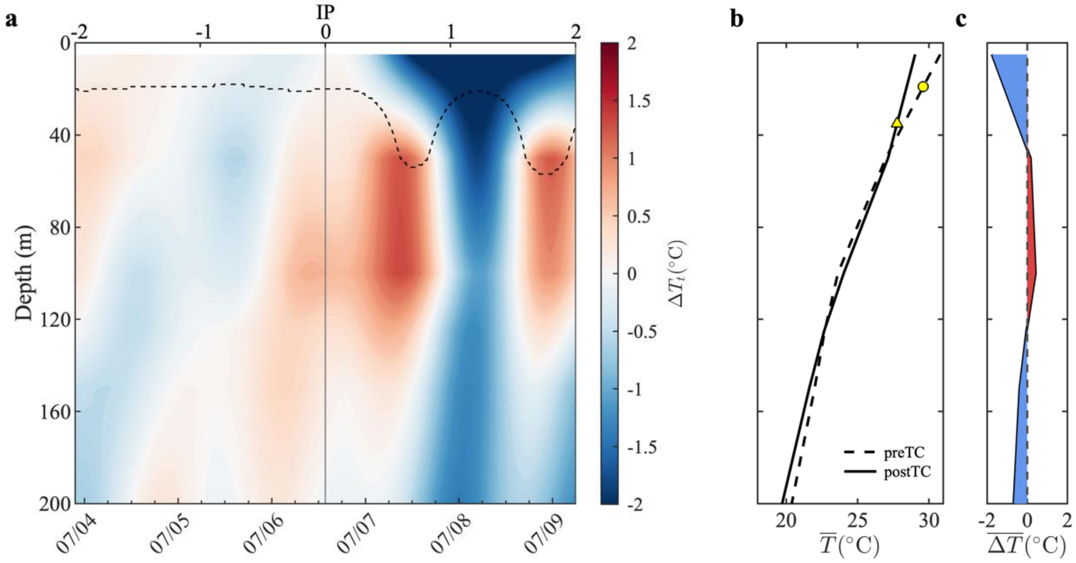

Supplementary Figure 4. Temperature data for Nepartak 2. The details are the same as those shown in Supplementary Figure 3.

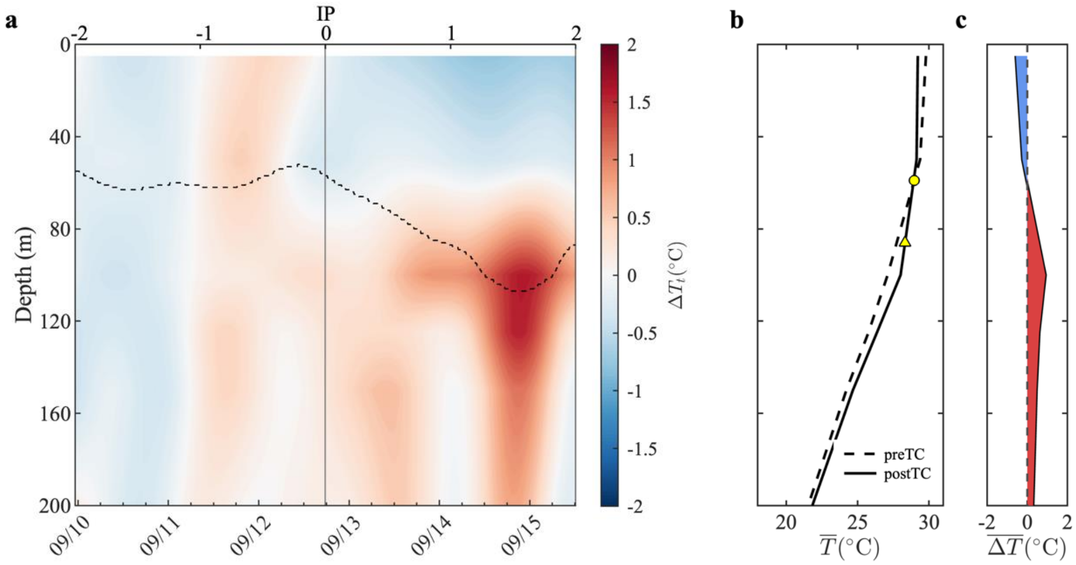

Supplementary Figure 5. Temperature data for Meranti 1. The details are the same as those shown in Supplementary Figure 3.

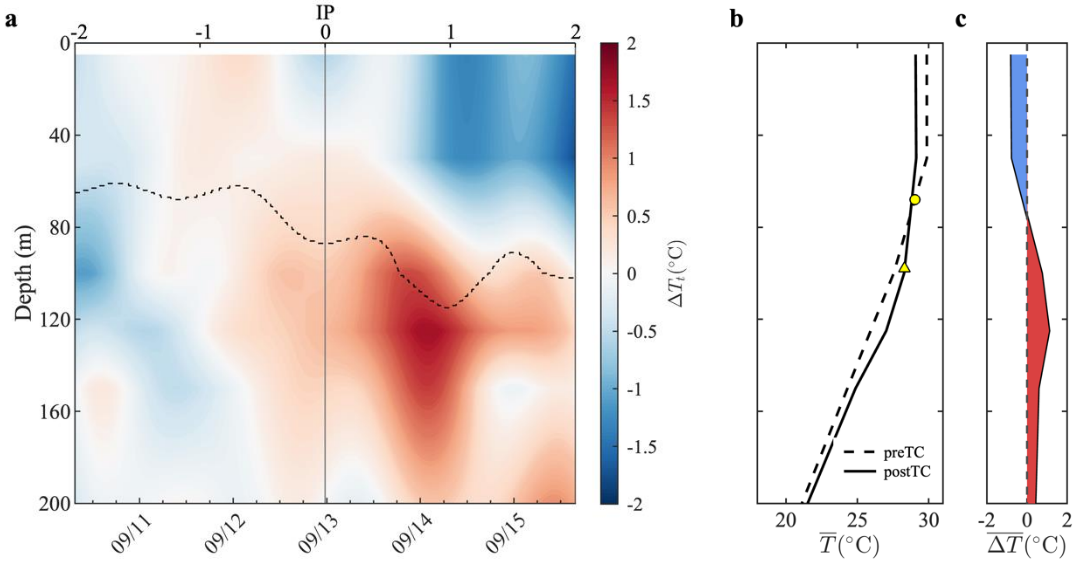

Supplementary Figure 6. Temperature data for Meranti 2. The details are the same as those shown in Supplementary Figure 3.

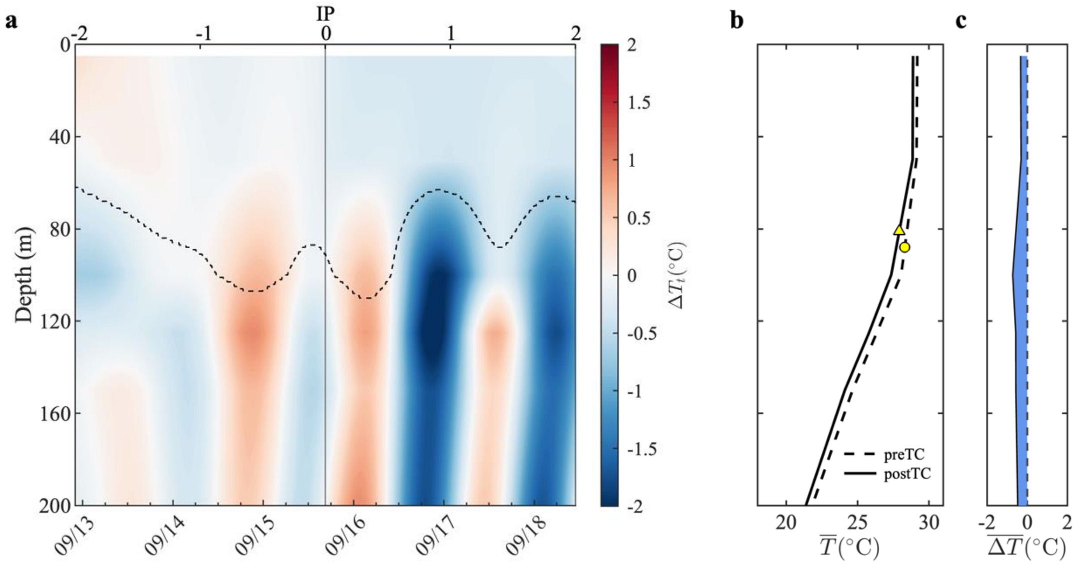

Supplementary Figure 7. Temperature data for Malakas 1. The details are the same as those shown in Supplementary Figure 3.

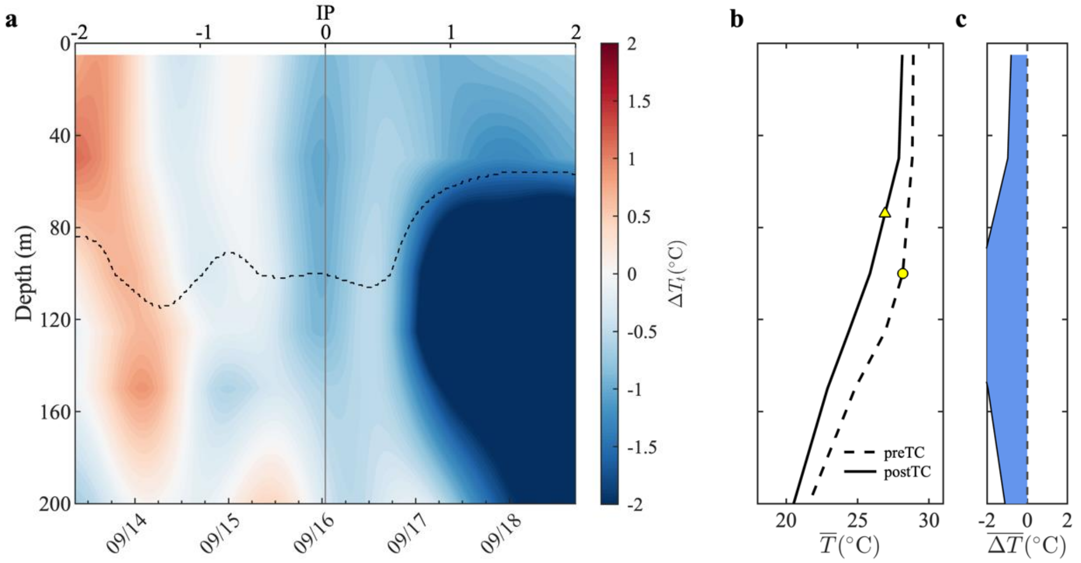

Supplementary Figure 8. Temperature data for Malakas 2. The details are the same as those shown in Supplementary Figure 3.

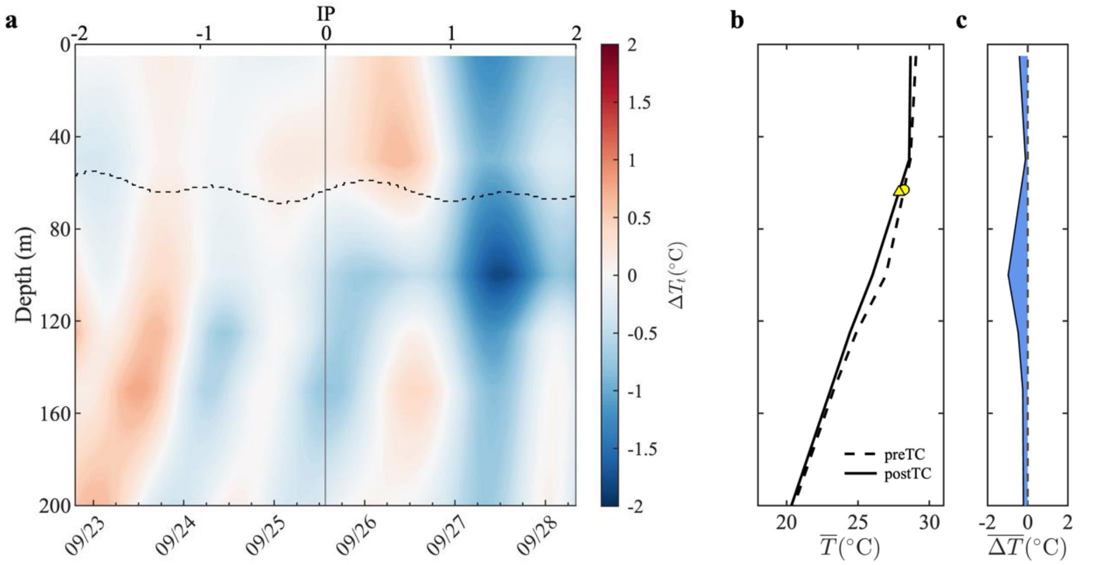

Supplementary Figure 9. Temperature data for Megi 1. The details are the same as those shown in Supplementary Figure 3.

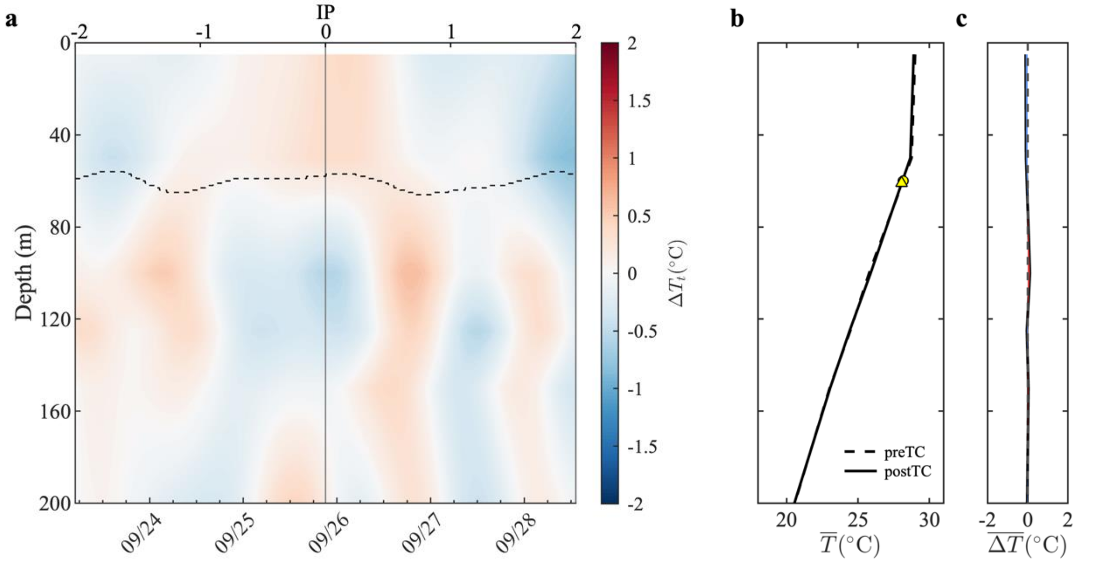

Supplementary Figure 10. Temperature data for Megi 2. The details are the same as those shown in Supplementary Figure 3.

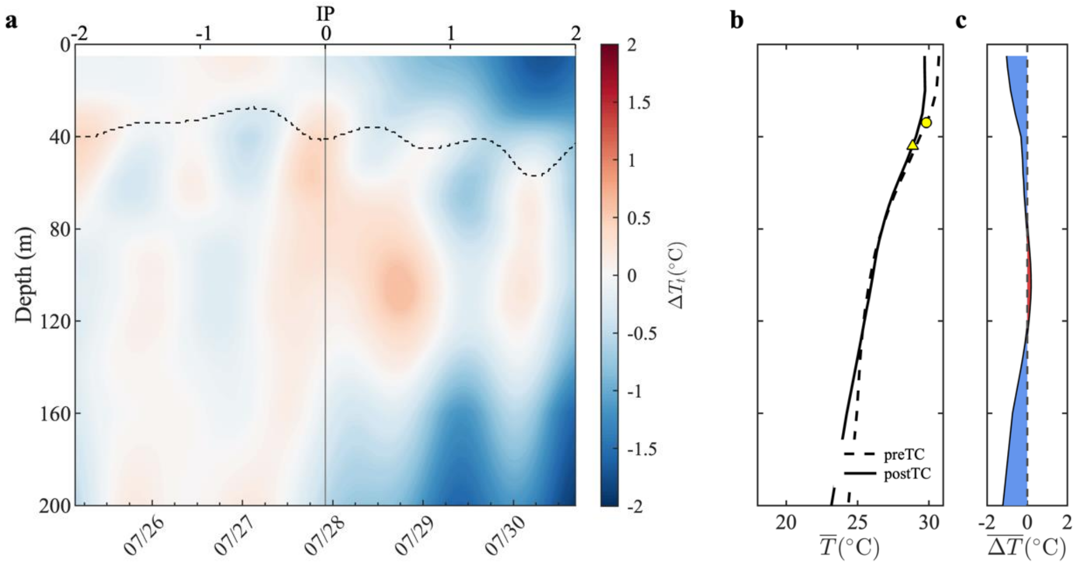

Supplementary Figure 11. Temperature data for Nesat 1. The details are the same as those shown in Supplementary Figure 3.

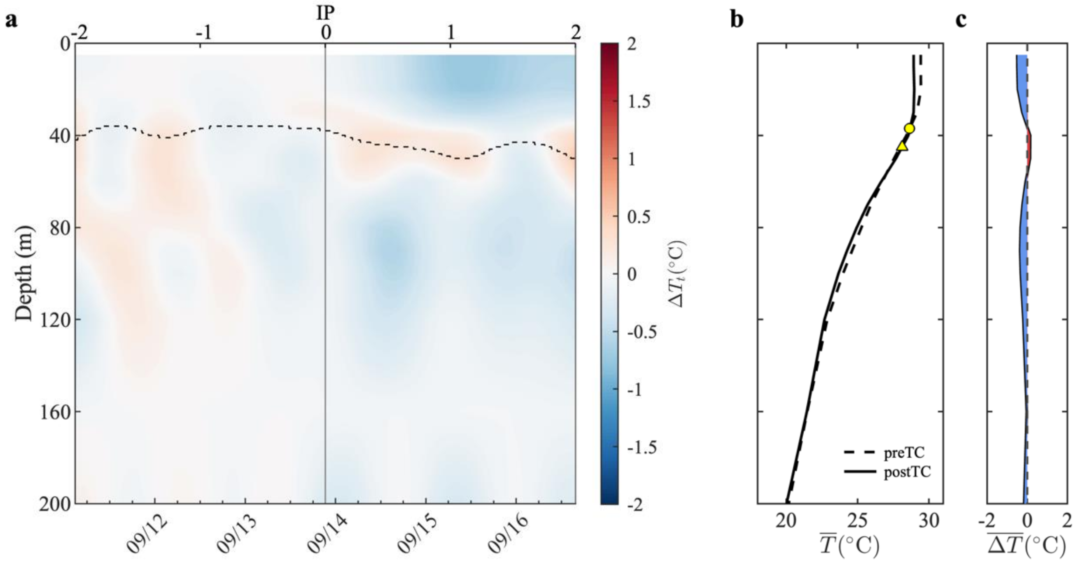

Supplementary Figure 12. Temperature data for Mangkhut 1. The details are the same as those shown in Supplementary Figure 3.

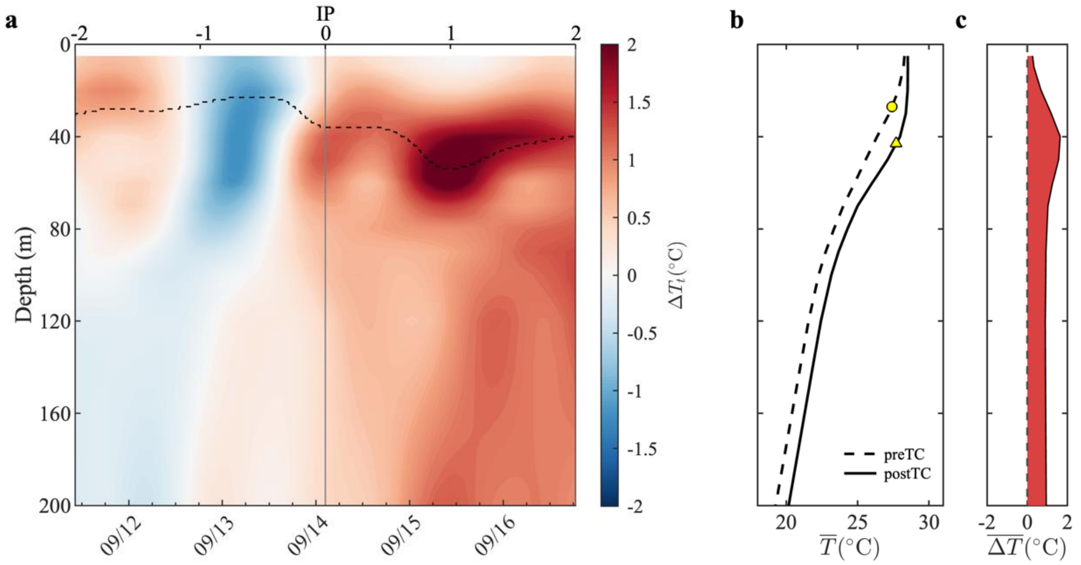

Supplementary Figure 13. Temperature data for Mangkhut 2. The details are the same as those shown in Supplementary Figure 3.

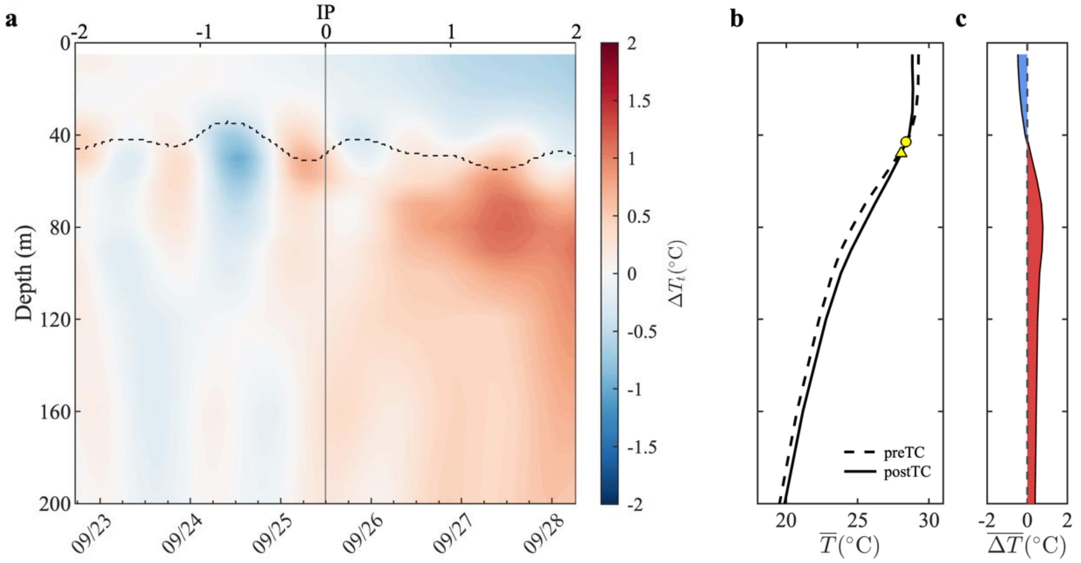

Supplementary Figure 14. Temperature data for Trami 1. The details are the same as those shown in Supplementary Figure 3.

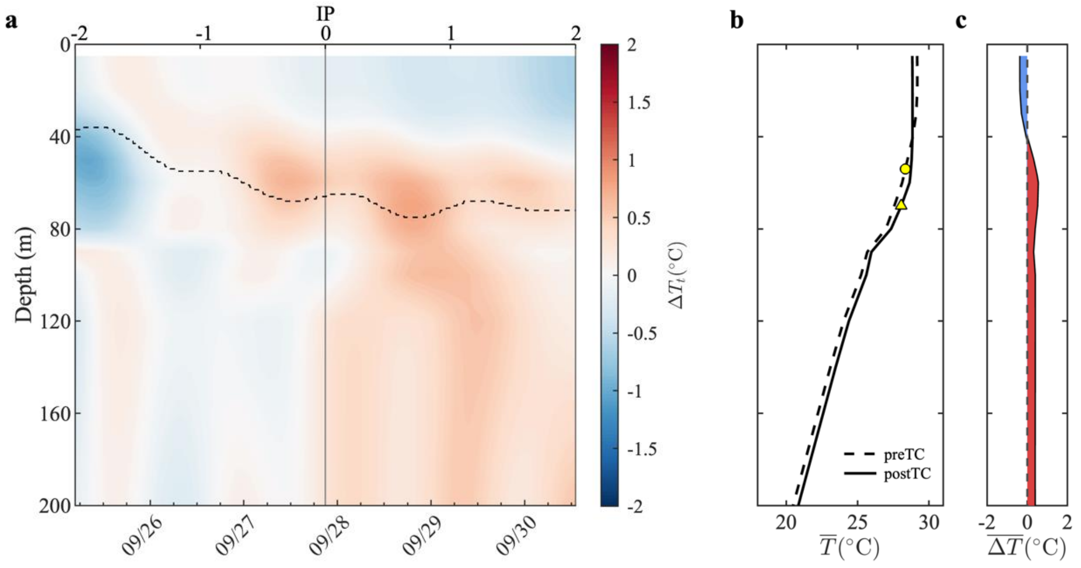

Supplementary Figure 15. Temperature data for Trami 2. The details are the same as those shown in Supplementary Figure 3.

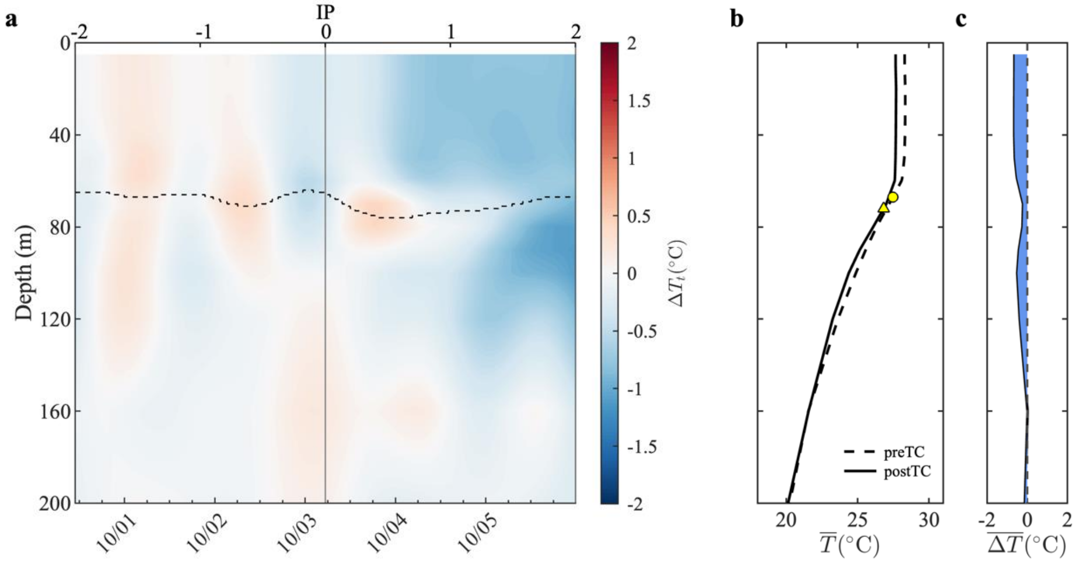

Supplementary Figure 16. Temperature data for Kongrey 1. The details are the same as those shown in Supplementary Figure 3.

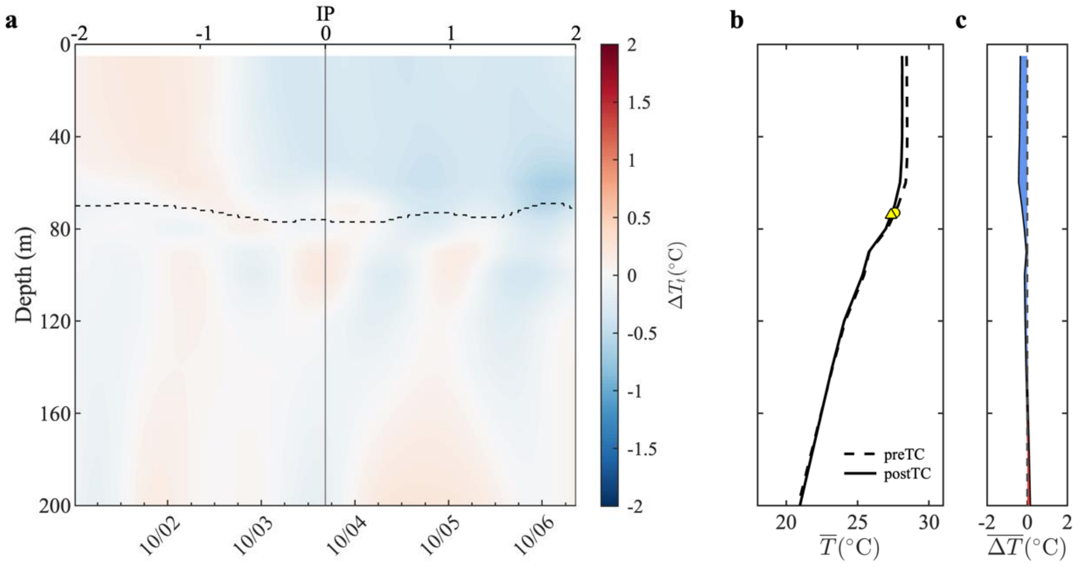

Supplementary Figure 17. Temperature data for Kongrey 2. The details are the same as those shown in Supplementary Figure 3.

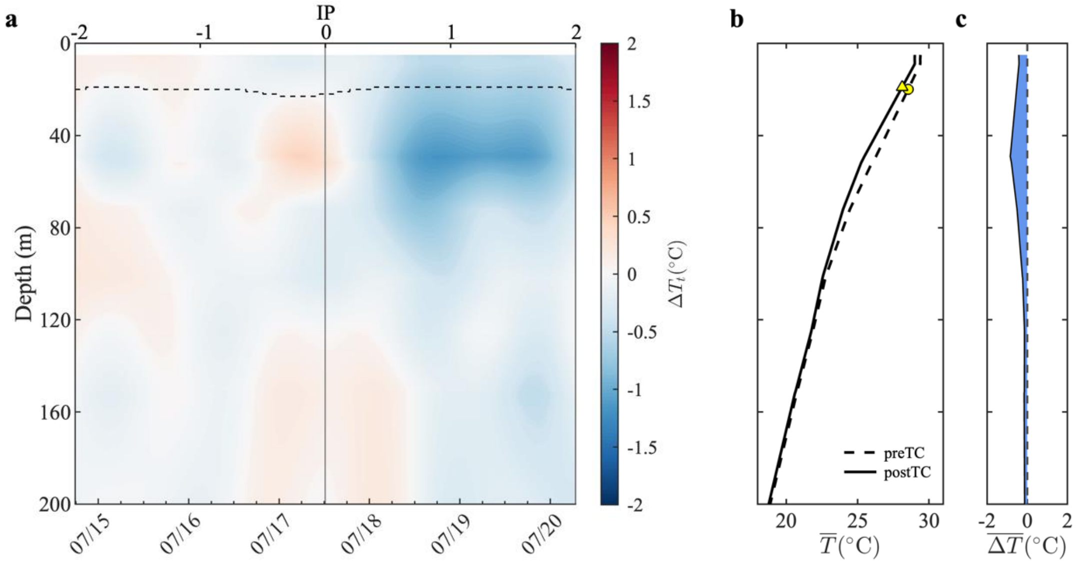

Supplementary Figure 18. Temperature data for Danas 1. The details are the same as those shown in Supplementary Figure 3.

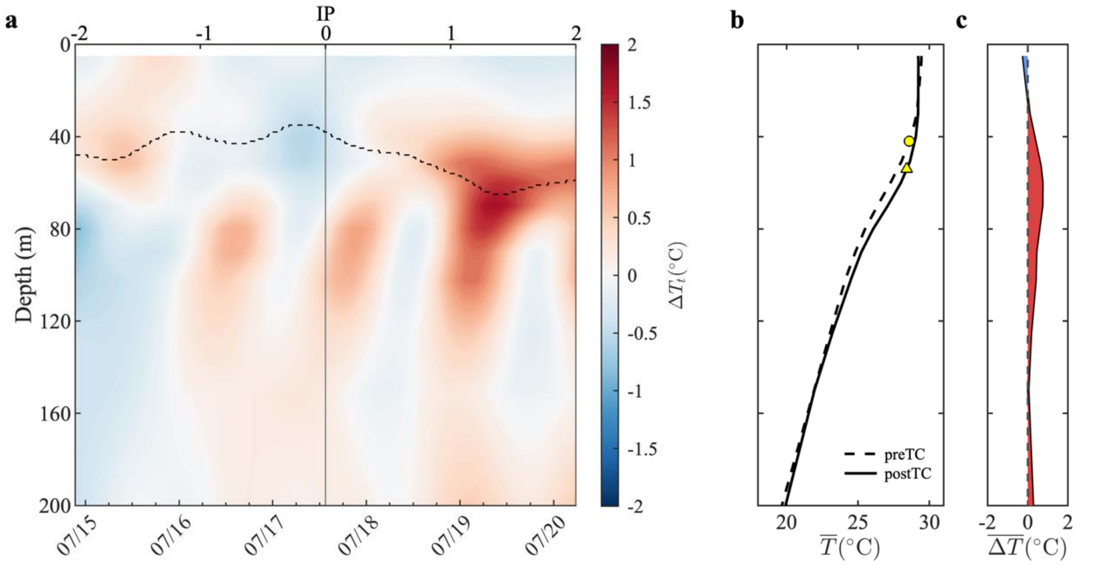

Supplementary Figure 19. Temperature data for Danas 2. The details are the same as those shown in Supplementary Figure 3.

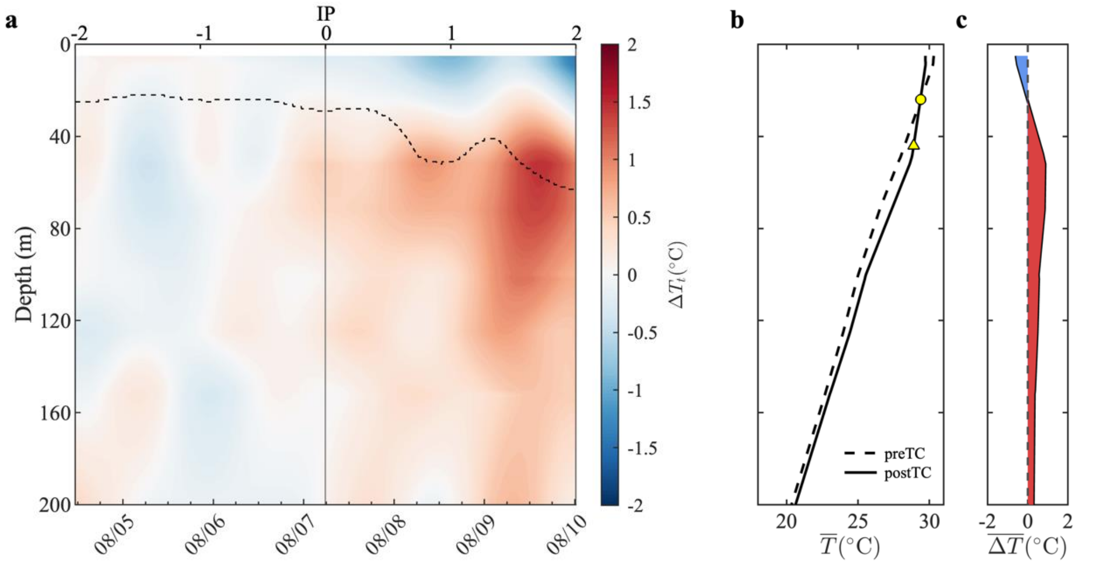

Supplementary Figure 20. Temperature data for Lekima 1. The details are the same as those shown in Supplementary Figure 3.

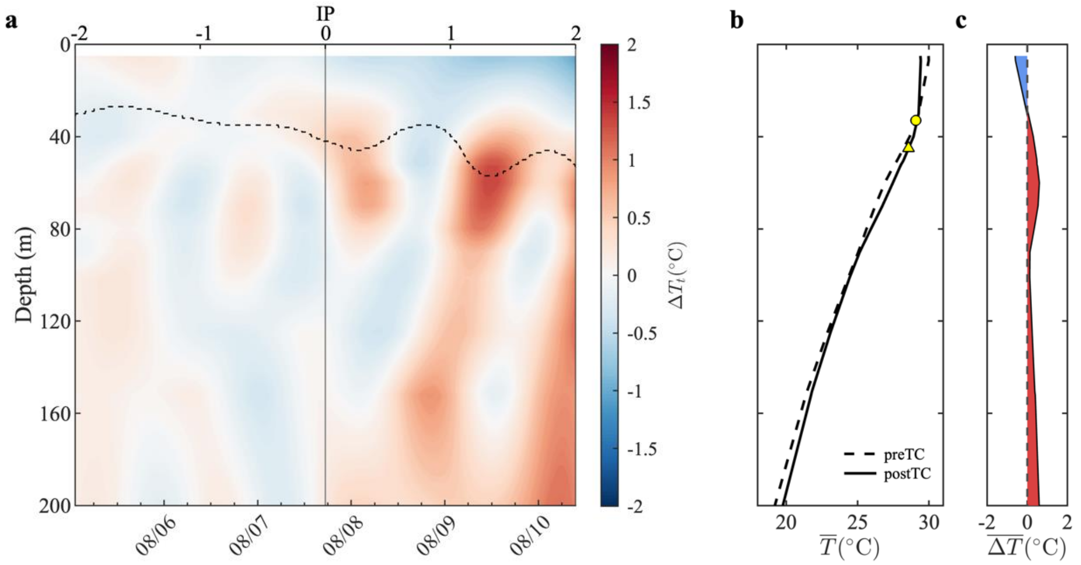

Supplementary Figure 21. Temperature data for Lekima 2. The details are the same as those shown in Supplementary Figure 3.

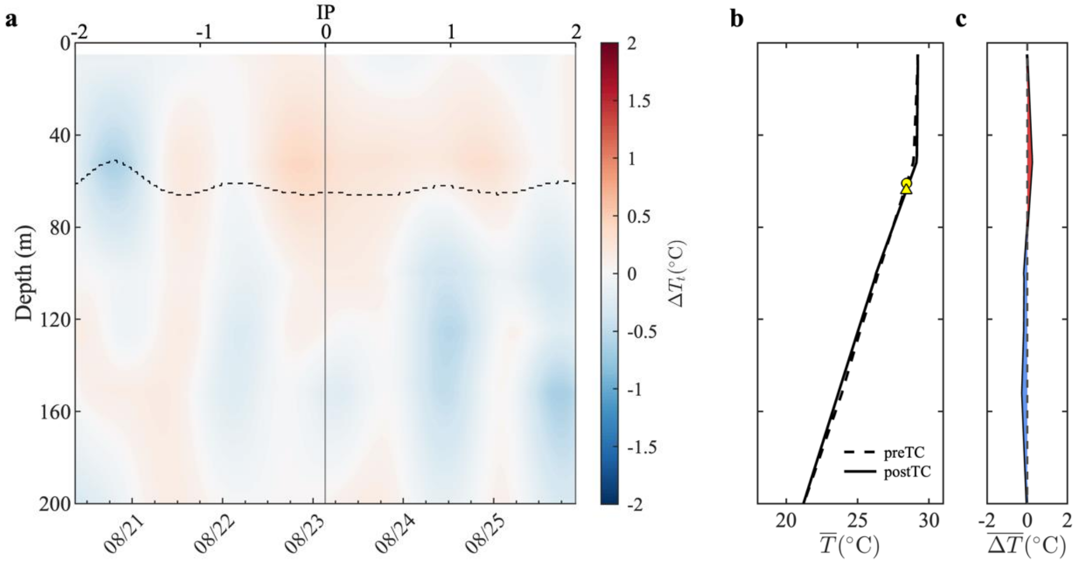

Supplementary Figure 22. Temperature data for Bailu 1. The details are the same as those shown in Supplementary Figure 3.

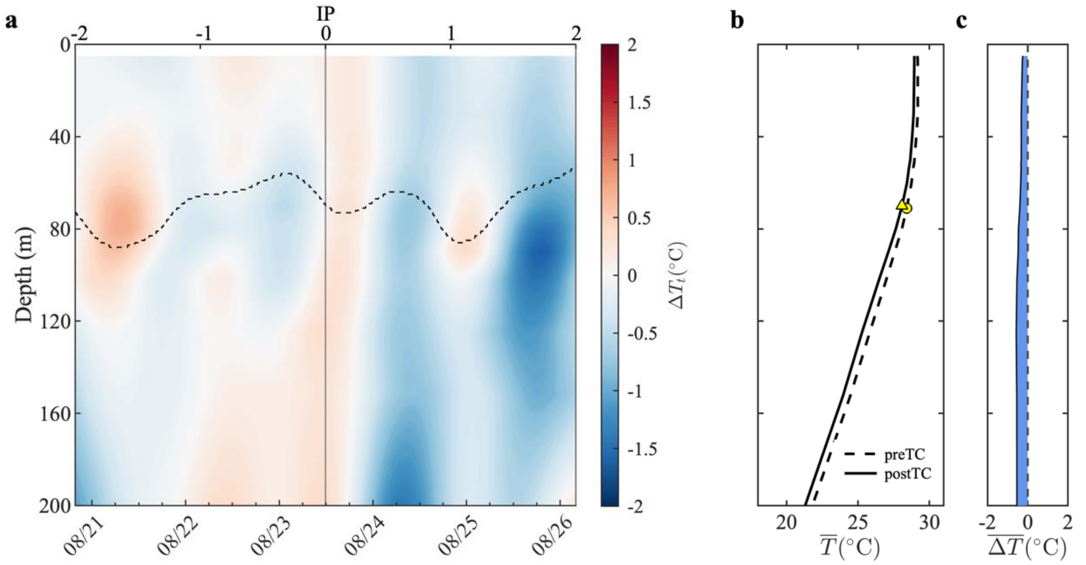

Supplementary Figure 23. Temperature data for Bailu 2. The details are the same as those shown in Supplementary Figure 3.

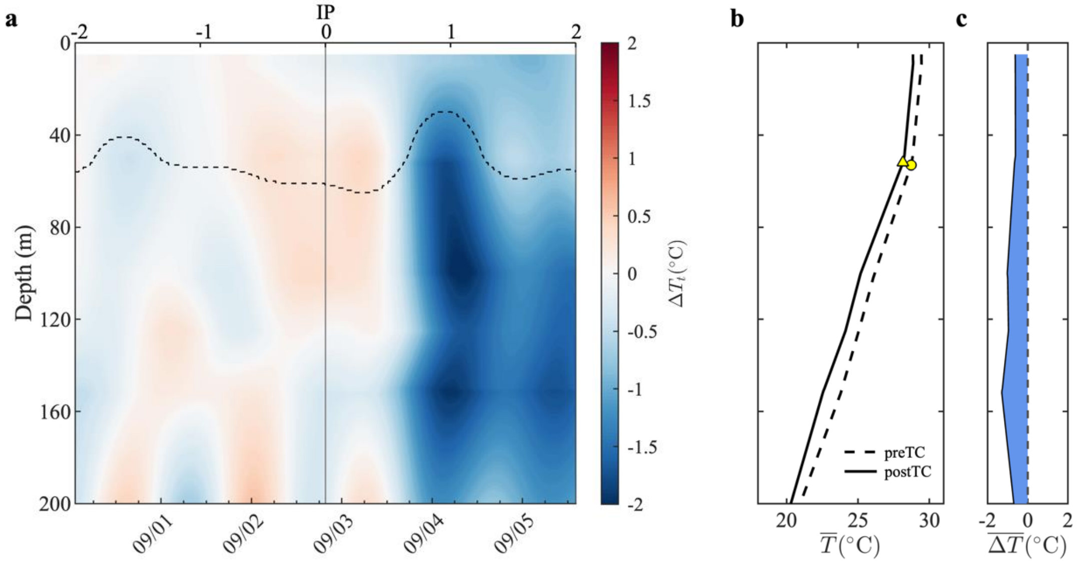

Supplementary Figure 24. Temperature data for Lingling 1. The details are the same as those shown in Supplementary Figure 3.

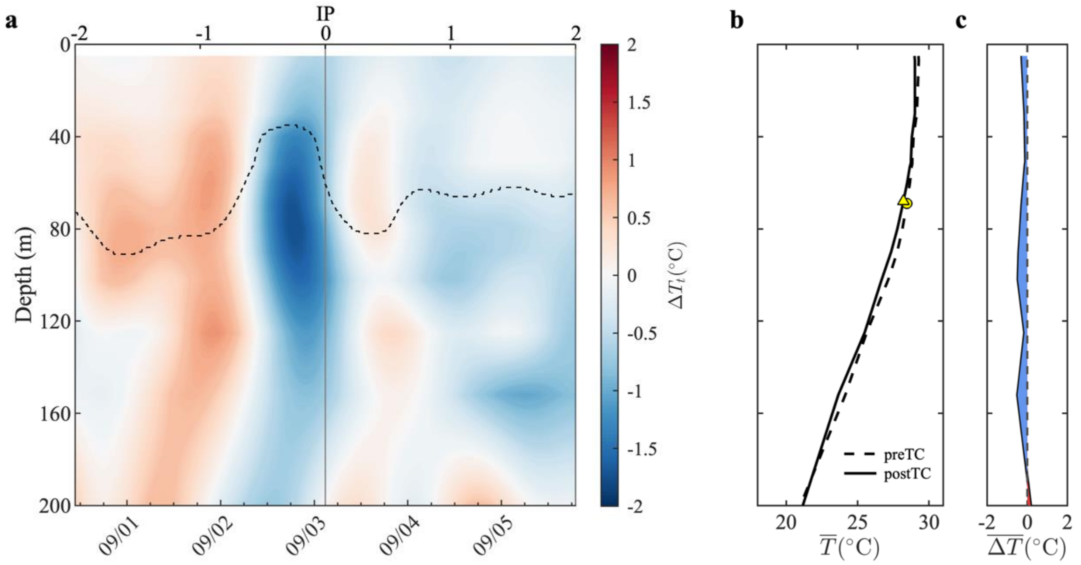

Supplementary Figure 25. Temperature data for Lingling 2. The details are the same as those shown in Supplementary Figure 3.

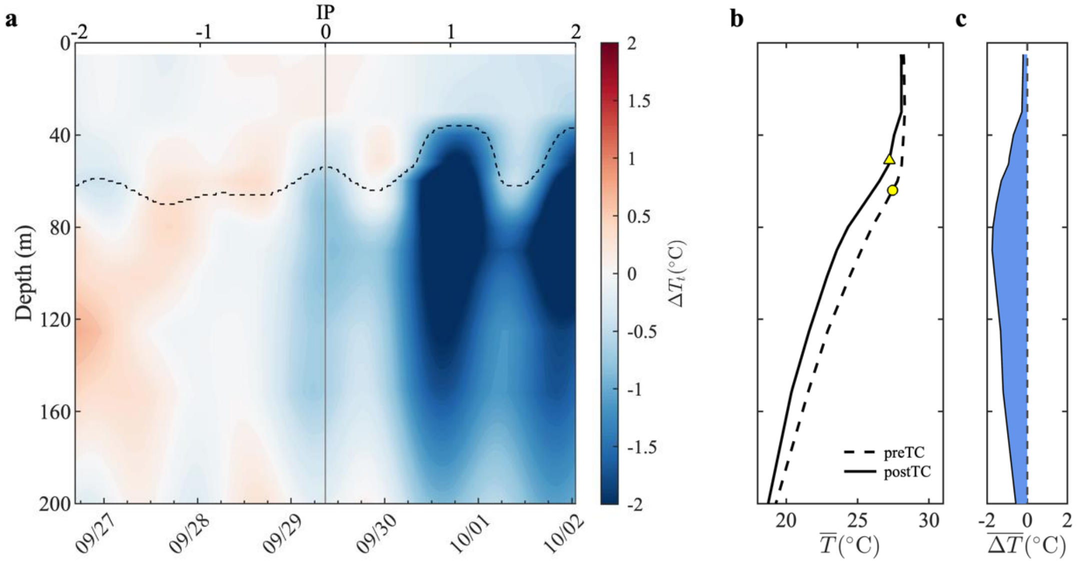

Supplementary Figure 26. Temperature data for Mitag 2. The details are the same as those shown in Supplementary Figure 3.

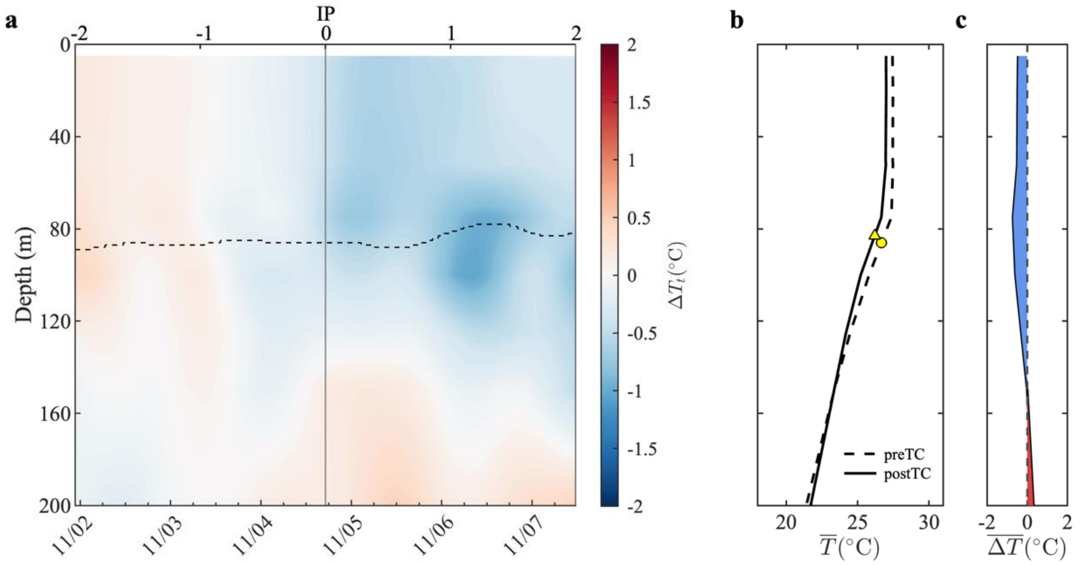

Supplementary Figure 27. Temperature data for Atsani 1. The details are the same as those shown in Supplementary Figure 3.

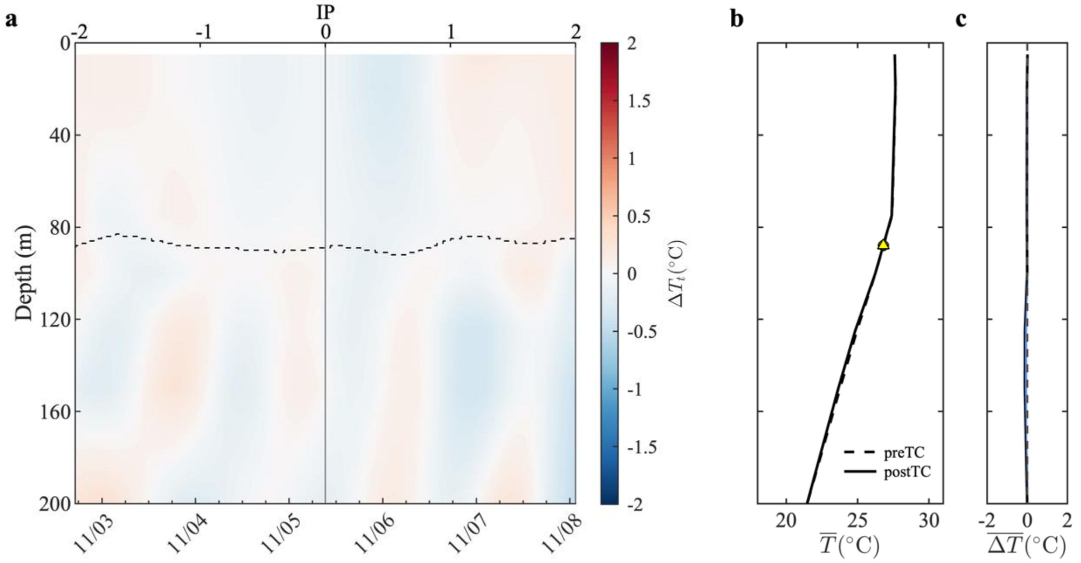

Supplementary Figure 28. Temperature data for Atsani 2. The details are the same as those shown in Supplementary Figure 3.

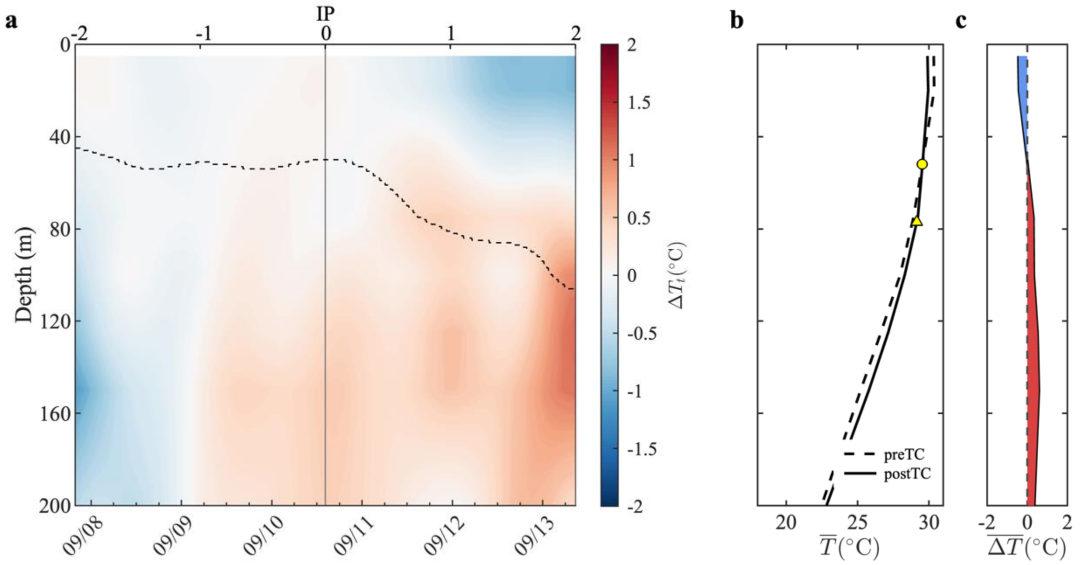

Supplementary Figure 29. Temperature data for Chanthu 1. The details are the same as those shown in Supplementary Figure 3.

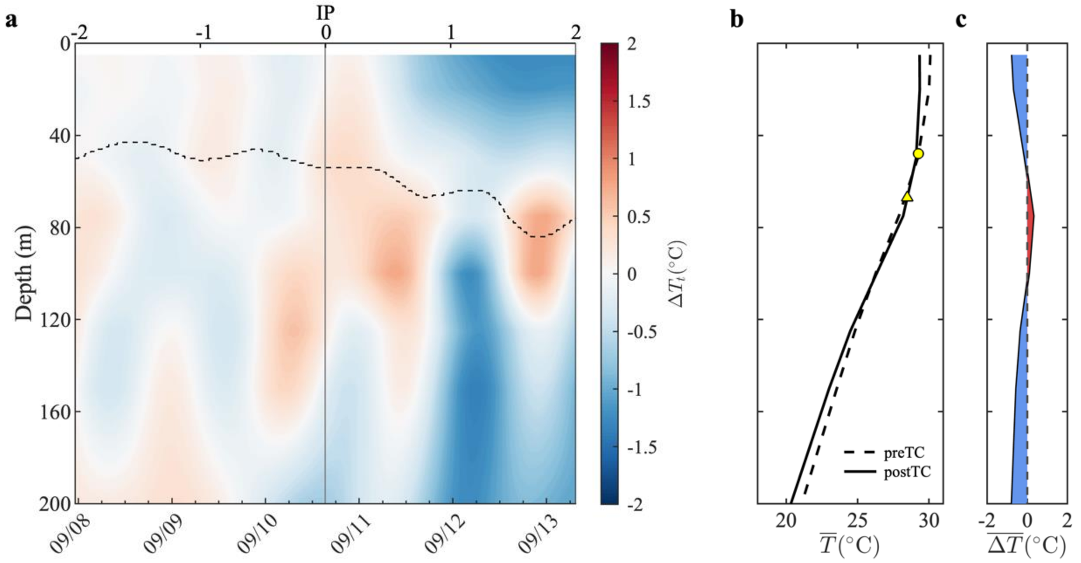

Supplementary Figure 30. Temperature data for Chanthu 2. The details are the same as those shown in Supplementary Figure 3.

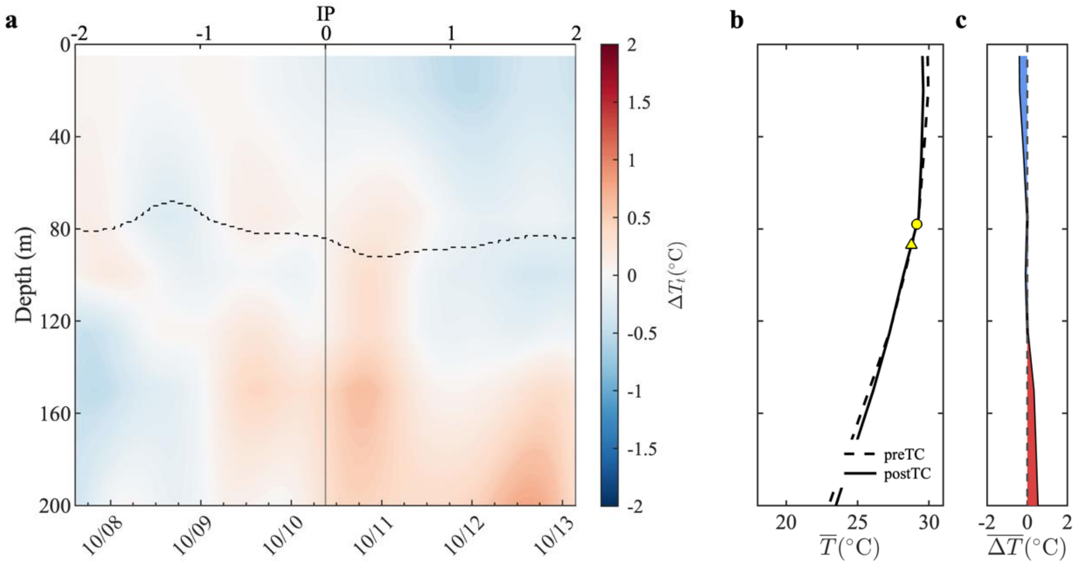

Supplementary Figure 31. Temperature data for Kompasu 1. The details are the same as those shown in Supplementary Figure 3.

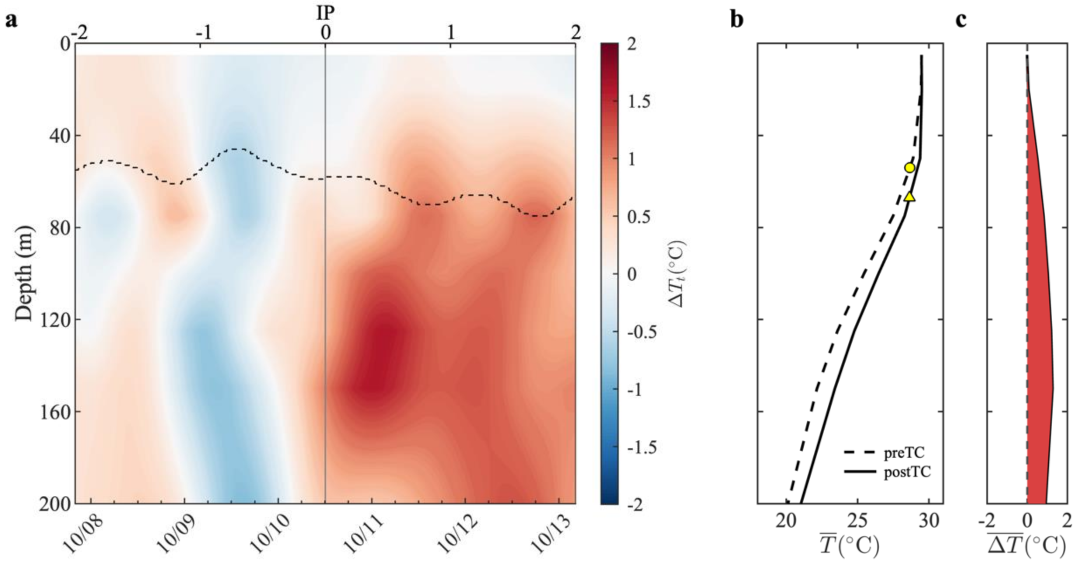

Supplementary Figure 32. Temperature data for Kompasu 2. The details are the same as those shown in Supplementary Figure 3.

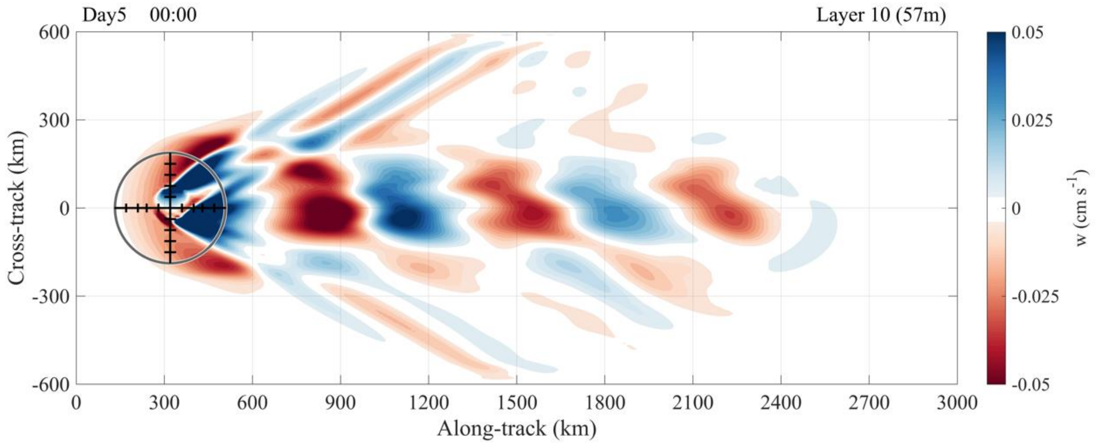

Supplementary Figure 33. Snapshot of *w* velocity component at layer 10 (57 m) on day 5 in Case A in the model experiments. Red shading indicates downwelling, and blue shading indicates upwelling. The gray circle denotes R_{34} of the TC. Each tick in the scale bar denotes $0.2R_{34}$ along four directions from the center of the TC.

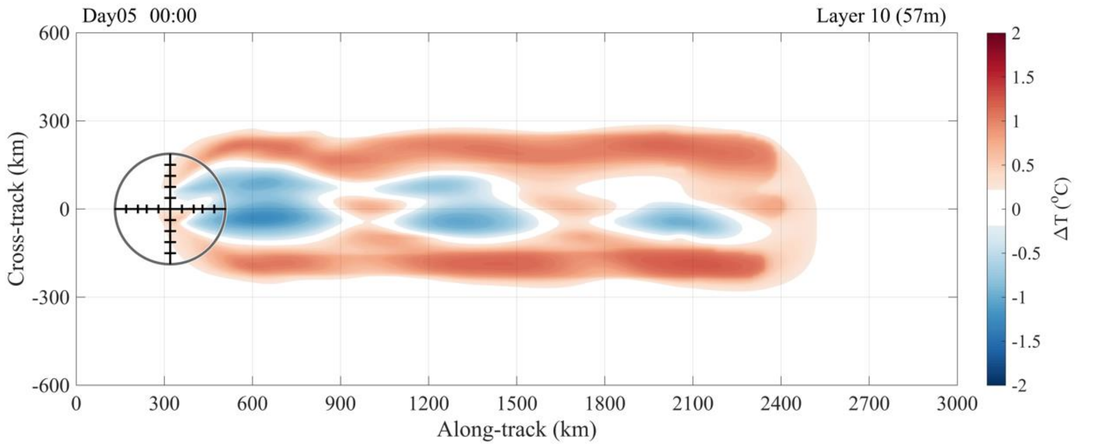

Supplementary Figure 34. Snapshot of ΔT at layer 10 (57 m) on day 5 in Case A in the model experiments. Red shading indicates temperature increases compared to the initial temperature; blue shading indicates a temperature decrease. The circle and scale bar provide the same information as those in Supplementary Figure 33.

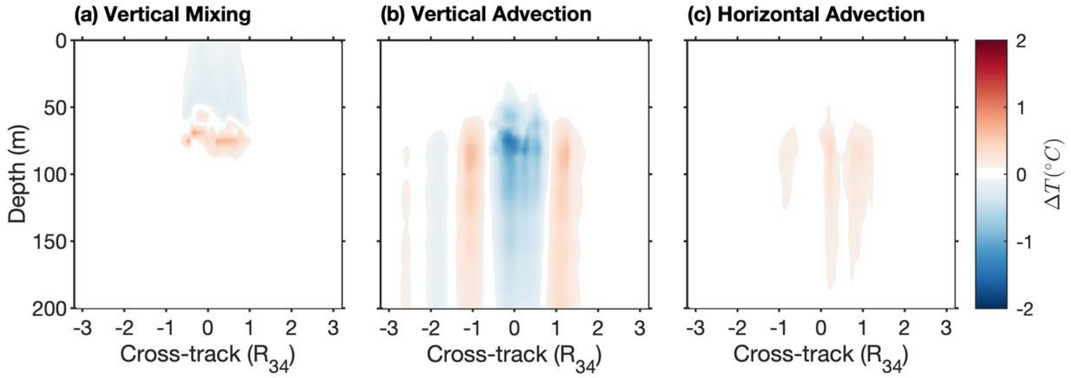

Supplementary Figure 35. Depth-crosstrack section of ΔT_t for the numerical experiment Case B. The temperature anomaly may be attributed to (**a**) vertical mixing, (**b**) vertical advection, and (**c**) horizontal advection.

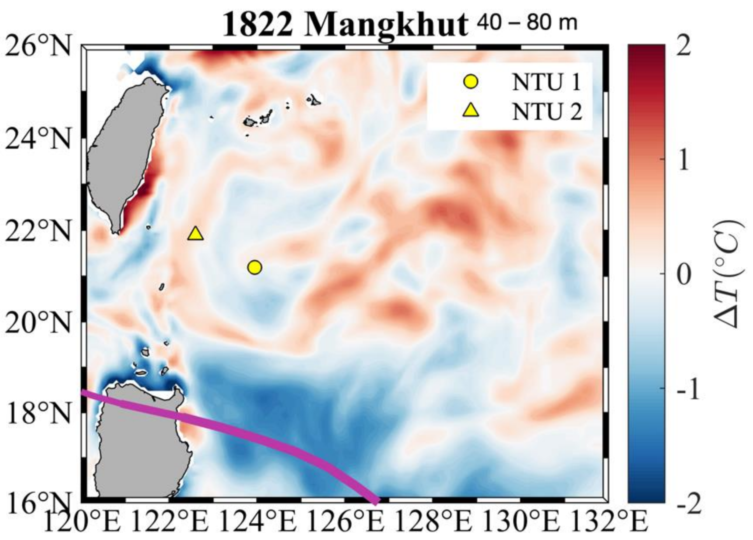

Supplementary Figure 36. Vertically averaged temperature changes before and after TC Mangkhut (2018) simulated by the Global Ocean Forecasting System (GOFS). We adopted GOFS 3.1 (https://www.hycom.org/), which is based on the Hybrid Coordinate Ocean Model and assimilated by the Navy Coupled Ocean Data Assimilation with the incorporation of observation data (S1, S2). The selected depths are based on the warming depths in Fig. 2b in the Main text. Red (blue) shading indicates warming (cooling). The TC track is marked in magenta, and the yellow circle and triangle denote the locations of the deployed buoys NTU1 and NTU2, respectively.

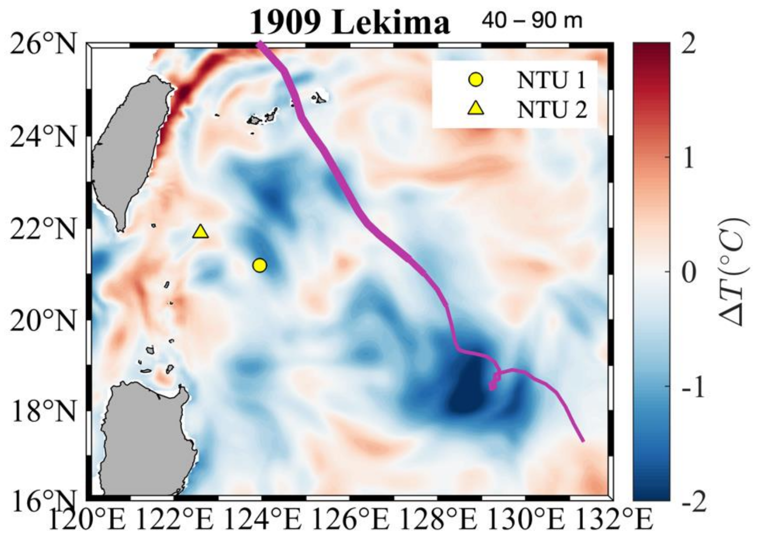

Supplementary Figure 37. Vertically averaged temperature changes before and after TC Lekima (2019) simulated by GOFS. The details are the same as those shown in Supplementary Figure 36.

Supplementary Table 1. Additional information for the sixteen TCs examined. The Saffir-Simpson Hurricane Scale (SSHS) is determined by calculating the average intensity during the period at which the buoy is within the $1.5R_{34}$ value of the TC, rather than its lifetime maximum intensity. TC translation speed is determined by calculating the average speed during the period at which the buoy is within the $1.5R_{34}$ value of the TC.

No.	Name	Buoy	SSHS	Speed ($m\ s^{-1}$)
1601	Nepartak	1	C5	5.8
		2	C3	4.4
1614	Meranti	1	C5	6.4
		2	C5	6.3
1616	Malakas	1	C3	5.9
		2	C3	5.8
1617	Megi	1	C3	5.9
		2	C2	6.0
1709	Nesat	1	C1	5.2
1822	Mangkhut	1	C4	7.6
		2	C4	7.7
1824	Trami	1	C2	2.5
		2	C2	4.0
1825	Kong-rey	1	C1	5.6
		2	C1	6.0
1905	Danas	1	TS	8.2
		2	TS	7.9
1909	Lekima	1	C4	4.8
		2	C4	5.0
1911	Bailu	1	TS	6.9
		2	TS	7.1
1913	Lingling	1	C2	3.2
		2	C2	3.1
1916	Mitag	2	C1	6.9
2020	Atsani	1	TS	5.9
		2	TS	5.5
2114	Chanthu	1	C4	4.8
		2	C4	5.0
2118	Kompasu	1	TS	5.9
		2	TS	6.5

Reference

S1. Cummings, J. A. Operational multivariate ocean data assimilation. *Quarterly Journal of the Royal Meteorological Society*, **131**, 3583–3604 (2005).

S2. Cummings, J. A. & Smedstad, O. M. Variational Data Assimilation for the Global Ocean. In: Park, S., Xu, L. (Eds.) Data Assimilation for Atmospheric, Oceanic and Hydrologic Applications. Vol. II. Springer, Berlin, Heidelberg. (2013).
